# Chip-to-chip photonic quantum teleportation over optical fibers of 12.3 km

**DOI:** 10.1038/s41377-025-01920-z

**Published:** 2025-07-09

**Authors:** Dongning Liu, Zhanping Jin, Jingyuan Liu, Xiaotong Zou, Xiaosong Ren, Hao Li, Lixing You, Xue Feng, Fang Liu, Kaiyu Cui, Yidong Huang, Wei Zhang

**Affiliations:** 1 Frontier Science Center for Quantum Information, State Key Laboratory of Low-Dimensional Quantum Physics, Beijing National Research Center for Information Science and Technology (BNRist), Electronic Engineering Department, Tsinghua University, Beijing, China; 2 National Key Laboratory of Materials for Integrated Circuits, Shanghai Institute of Microsystem and Information Technology, Chinese Academy of Sciences, Shanghai, China; 3 Beijing Academy of Quantum Information Sciences, Beijing, China

**Keywords:** Optical techniques, Quantum optics

## Abstract

Quantum teleportation is a crucial function in quantum networks. The implementation of photonic quantum teleportation could be highly simplified by quantum photonic circuits. To extend chip-to-chip teleportation distance, more effort is needed on both chip design and system implementation. In this work, we demonstrate a time-bin-based chip-to-chip photonic quantum teleportation over optical fibers under the scenario of a star-topology quantum network. Three quantum photonic circuits are designed and fabricated on a single chip, each serving specific functions: heralded single-photon generation at the user node, entangled photon pair generation and BSM at the relay node, and projective measurement of the teleported photons at the central node. The unbalanced Mach–Zehnder interferometers (UMZI) for time-bin encoding in these quantum photonic circuits are optimized to reduce insertion losses and suppress noise photons generated on the chip. Besides, an active feedback system is employed to suppress the impact of fiber length fluctuation between the circuits, achieving a stable quantum interference for the BSM in the relay node. As a result, a photonic quantum teleportation over optical fibers of 12.3 km is achieved based on these quantum photonic circuits, showing the potential of chip integration for the development of quantum networks.

## Abstract (web summary)

This work achieves experimental chip-to-chip quantum teleportation with a transmission distance of 12.3 km over optical fibers, showing the potential of chip integration for the development of quantum networks.

## Graphical abstract

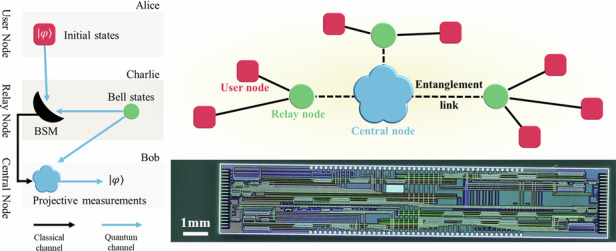

## Introduction

Quantum teleportation transfers quantum states through pre-established entanglement, providing a way to deliver quantum information without transmitting its physical carrier. Since it was proposed in 1993^1^, quantum teleportation has been realized in various quantum systems^2–7^. Photons, as “flying” qubits, are good carriers of quantum information to achieve long-distance quantum teleportation. The photonic quantum teleportation has been demonstrated over indoor fiber spools of 102 km^8^, metropolitan fiber cables^9–13^, and free space between satellite and ground^14^. Quantum teleportation is an essential function in the quantum network with a star topology^9^. In this network, a central node serves as a quantum information processor, and many user nodes send their quantum information to the central node as input for quantum information processing. If the central node is far from the users, quantum information of the users can be carried by photons and transmitted by low-loss optical channels such as optical fibers. However, directly transmitting photons is impractical in some scenarios. For instance, if optical channels to the central node for quantum information transmission are scarce in an area, it is uneconomical, or even impossible, for each user in that area to transmit photons directly. Besides, if the wavelength of the photons (for instance, near 780 nm photons generated by atomic ensemble quantum memories^15,16^) does not meet the requirements of the optical channels (such as the telecommunication band used for low-loss transmission in optical fibers), the distance of directly transmitting photons would also be limited. In such scenarios, a quantum relay configuration^17,18^ can be employed to realize quantum information transmission. In this configuration, the user node only needs to send photons to a nearby relay node that shares prior-distributed entanglement resources with the central node. The quantum information is then teleported from the relay node to the central node, greatly reducing the required transmission distance for the photons and providing a robust alternative for quantum information transmission. Additionally, photonic devices for switching and routing could be set in the relay nodes to select a specific user node sending its quantum information, enabling multi-user access to the central node of the quantum network. It is worth noting that perfect quantum memories and entanglement distribution are required in the ideal quantum relay configuration, which are still unavailable currently. Experimental demonstration of quantum teleportation in a quantum relay configuration is usually demonstrated by probabilistic quantum light sources.

In recent years, quantum photonic circuits have developed rapidly. By integrating multiple optical components onto a single chip, compact and stable integrated quantum photonic systems can be developed to realize complicated and large-scale quantum information functions, such as complicated entanglement state generation^19–22^, quantum communication^23–26^, quantum computation and simulation^27–29^, and so on. It can be expected that the physical implementation of photonic quantum teleportation can also be highly simplified by integration technologies of quantum photonic circuits. Previous works have demonstrated the feasibility of quantum teleportation between chips, using path-encoded quantum states on the quantum photonic chips, which are transferred to polarization-encoded quantum states in optical fibers between them. The transmission distance in these quantum teleportation experiments is about 10 m^30,31^, far from the requirement of quantum networks. More effort is needed to extend the distance of quantum teleportation between chips, including chip design and system implementation.

In this work, we demonstrate a chip-to-chip photonic quantum teleportation over optical fibers under the scenario of a star-topology quantum network. To ensure robust quantum teleportation, we employ time-bin encoded quantum states, which are particularly effective in resisting fiber-induced fluctuations. Three quantum photonic circuits are designed and fabricated on a single chip, each serving specific functions: heralded single-photon generation at the user node, entangled photon pair generation and Bell state measurement (BSM) at the relay node, and projective measurement of the teleported photons at the central node. The unbalanced Mach–Zehnder interferometers (UMZI) for time-bin encoding in these quantum photonic circuits are optimized to reduce insertion losses and suppress noise photons generated on the chip. Besides, an active feedback system is employed to suppress the impact of fiber length fluctuation between the circuits, achieving a stable quantum interference for BSM in the relay node. As a result, the teleportation distance over optical fibers is 12.3 km in this work, showing the feasibility of chip-to-chip photonic quantum teleportation to be implemented in metropolitan fiber networks.

## Results

### Quantum photonic circuits for the user, relay, and central nodes

**Fig. 1:**
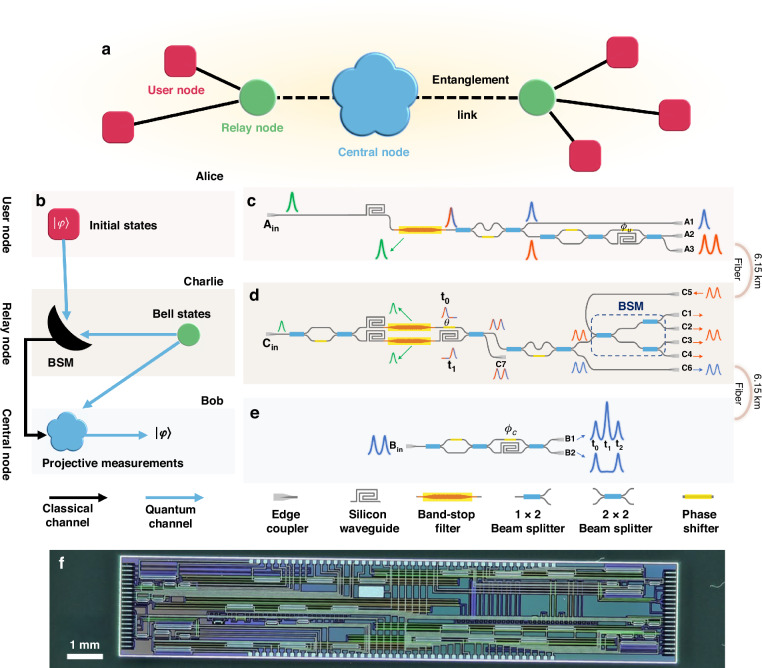
**a** Scenario of a star-topology quantum network. **b** Functions of the photonic quantum teleportation involving the user node, relay node, and central node. **c**–**e** Designs of the silicon photonic circuits for the user, relay, and central nodes, implementing the main functions required for quantum teleportation. **f** Photograph of the fabricated silicon photonic chip, which integrates all three quantum photonic circuits. Its size is 3 mm × 16 mm

A scenario of photonic quantum teleportation in a star-topology quantum network is considered in this work, which is shown in Fig. 1a. The user node sends photons with specific quantum states to a relay node. The relay node generates photon pairs with an entangled Bell state, one photon from each pair used for joint BSMs with photons from the user node, and the other photon sent to the central node. In the central node, the state of teleported photons from the relay node is transformed according to the BSM results, recovering the quantum states of the photons sent by the user node. In this work, we demonstrate it in a simplified way, in which the state of a photon sent to the central node is determined by the BSM result. The quality of the state indicates whether the quantum teleportation process is successful or not. Functions of the user node, the relay node, and the center node are shown in Fig. 1b. To characterize the states of photons sent to the central node, a projective measurement system is set at the central node to perform quantum state tomography (QST). Three silicon photonic quantum circuits are designed to realize the three nodes, respectively, which are depicted in Fig. 1c–e.

The quantum photonic circuit of the user node generates heralded, arbitrary time-bin encoded single-photon states, as shown in Fig. 1c. It needs a pulsed pump light, which is coupled into the chip through the left-side edge coupler. Correlated photon pairs are generated via spontaneous four-wave mixing (SFWM) in a long single-mode silicon waveguide with a cross-section of 500 nm × 220 nm and a length of 1 cm. Subsequently, a waveguide Bragg grating (WBG) band-stop filter^32–35^ is used to filter out the pump light, preventing noise photons generated by the residual pump light^36–38^. An unbalanced Mach–Zehnder interferometer with a free spectral range (FSR) of 1600 GHz (referred to as the 1600 GHz-UMZI hereafter) separates the signal and idler photons in the generated photon pair into two paths. The signal photons are output from the port A1 directly, which are used to generate heralding signals. The idler photons enter an unbalanced Mach–Zehnder interferometer with an optical path difference of 400 ps (referred to as the 400 ps-UMZI hereafter), which generates the time-bin encoded single photon state $\left| \psi \right\rangle = \alpha \left| 0 \right\rangle + \beta \mathrm{e}^{i\phi_u} \left| 1 \right\rangle$. When the photons pass through the short arm of the 400 ps-UMZI, they are encoded into the state of the earlier time-bin $|0\rangle$, while they are encoded into the state of the later time-bin $|1\rangle$ if they pass through the long arm. A thermo-optic phase shifter (TOPS) in the 400 ps-UMZI adjusts the phase ${\phi }_{u}$ in the superposition state. It is worth noting that the length of the long arm in the 400 ps-UMZI is as long as 3.2 cm. Reducing the transmission loss of the long arm and balancing the two arms are crucial for the chip-to-chip photonic teleportation experiment. Two designs are applied. First, the straight parts of the long arm are realized by rib waveguides with a rib width of 1.5 μm and a depth of 130 nm. Their propagation losses are about 0.2 dB/cm. The transition losses between the rib waveguides and single-mode strip waveguides are negligible through optimizing the transition section. Second, a variable beam-splitter (VBS) formed by a balanced MZI is used as the first beam-splitter in the 400 ps-UMZI, adjusting the amplitudes of $|0\rangle$ and $|1\rangle$ in the state. In this work, this type of UMZIs with VBSs is applied in several places, which is referred to VBS-UMZI hereafter. Details of the low-loss shallow rib waveguides and the WBG band-stop filter are introduced in the Supplementary Information S1.

The quantum photonic circuit of the relay node generates photon pairs of time-bin encoded entangled Bell states, performs BSM between the idler photons of the photon pairs and the received photons from the user node, and sends the signal photons to the central node, as shown in Fig. 1d. It also needs a pulsed pump light, which is coupled into the chip through the edge coupler and injected into a VBS-UMZI. The VBS splits the pump light into the two arms. The short arm includes a single-mode silicon waveguide with a length of 1 cm and a WBG band-stop filter. The pump light generates photon pairs with a biphoton state via SFWM in this waveguide, then suppressed by the WBG band-stop filter to avoid additional noise photons in the following photonic circuit. The long arm also includes a single-mode silicon waveguide with a length of 1 cm and a WBG band-stop filter to generate photon pairs with a biphoton state. Additionally, the long arm has a delay waveguide of 400 ps. The VBS-UMZI has two output ports, one of which is used to superpose the biphoton states generated in the short arm and long arm coherently with a time difference of 400 ps, forming the time-bin encoded entangled state $|\Phi \rangle =\frac{1}{\sqrt{2}}(|{0}_{s}{0}_{i}\rangle +{e}^{i\theta }|{1}_{s}{1}_{i}\rangle$. Here, “0” and “1” also denote the earlier and later time bins. “*s*” and “*i*” denote the signal and idler photons in photon pairs, respectively. A TOPS is located at the short arm, which is used to control the phase *θ* in the state. Specifically, the Bell state $\left|{\Phi }^{\pm }\right\rangle$ can be generated if *θ* is adjusted to 0 or *π*. Then, the signal and idler photons of the photon pairs with the entangled Bell state are separated by a 1600 GHz-UMZI. The signal photons (blue photons in Fig. 1d) are output from port C6 and sent to the central node for entanglement distribution. The idler photons (red photons in Fig. 1d) are sent to the on-chip Bell state analyzer in the quantum photonic circuit, which is formed by cascaded beam-splitters. The photons from the user node also inject into the Bell state analyzer through port C5. For the time-bin encoded biphoton states, the Bell state analyzer based on cascaded beam-splitters can distinguish $\left|{\Psi }^{\pm }\right\rangle =\frac{1}{\sqrt{2}}(|{0,1}\rangle \pm |{1,0}\rangle$ between the four Bell states. By analyzing the coincidence counts at the four output ports (C1–C4), the results of BSM can be obtained and sent to the central node. It is worth noting that the signal and idler photons are also output from the other output port of the VBS-UMZI, which is coupled out of the chip directly from the port C7 and used as a reference signal to stabilize the synchronization between the user node and the relay node.

The quantum photonic circuit of the central node characterizes the state of the photons sent from the relay node when they are post-selected by the BSM results. To demonstrate the performance of photonic quantum teleportation, a VBS-UMZI for projective measurements of time-bin encoded single-photon states is designed in the circuit of the central node, with its phase denoted as ${\phi }_{c}$, as shown in Fig. 1e. The photons from the relay node are coupled into the chip through the left-side edge coupler. By analyzing the photons output from B1 and B2 ports, projective measurements and QST are carried out, which are described in the “Materials and methods”. It is worth noting that in the relay node and central node, the long arms of VBS-UMZIs are also realized by low-loss shallow rib waveguides.

The three quantum photonic circuits are integrated into a silicon photonic chip with a size of 3 mm × 16 mm. The chip is fabricated by standard processes of silicon photonics compatible with complementary metal oxide semiconductor (CMOS) processes (Advanced Micro Foundry, Singapore). Figure 1f is the photograph of the chip. The inputs and outputs of the pump lights and generated photons are realized by the optical coupling between a fiber array and edge couplers on the chip. The chip is packaged on a thermoelectric cooler (TEC) to achieve a temperature stability of ±0.002 °C. Details on the insertion losses of the chip are provided in the Supplementary Information S2.

### Experimental system for photonic quantum teleportation

**Fig. 2:**
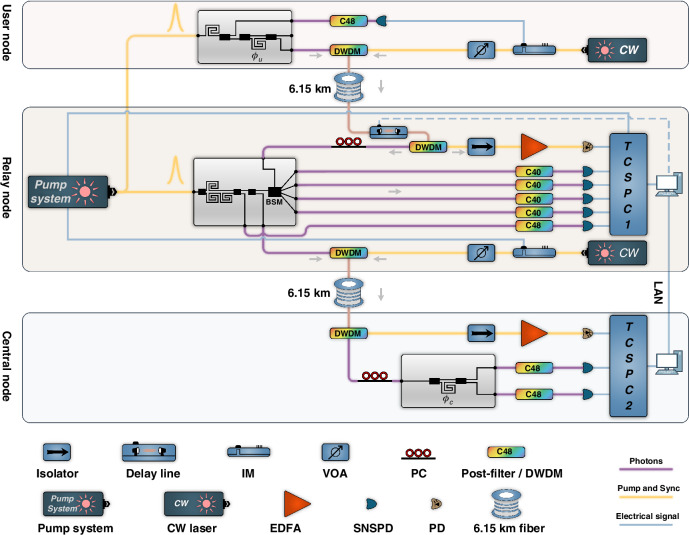
Experimental system of the chip-to-chip photonic quantum teleportation over an optical fiber of 12.3 km. The system includes time-bin encoded single-photon state generation at the user node, time-bin encoded entangled Bell state generation and BSM at the relay node, and projective measurements at the central node. The co-propagation of quantum signals and classical synchronization signals over optical fibers ensures the stable synchronization of photon arrival times in the fiber links. IM intensity modulator, VOA variable optical attenuator, PC polarization controller, DWDM dense wavelength division multiplexer, CW continuous wave, EDFA erbium-doped fiber amplifier, SNSPD superconducting nanowire single-photon detector, PD photodetector, LAN local area network

We use the packaged chip to perform the experiment of chip-to-chip photonic quantum teleportation over optical fibers of 12.3 km. The experimental system is shown in Fig. 2. In the user node, the quantum photonic circuit generates heralded time-bin encoded single-photon states. The wavelengths of the pump light, signal photons, and idler photons are 1542.14 nm, 1538.98 nm, and 1545.32 nm, respectively, corresponding to the International Telecommunication Union (ITU) channels of C44, C48, and C40. The pump light of the user node is generated by a pulsed fiber laser system with a repetition rate of 100 MHz. In the quantum photonic circuit of the user node, photon pairs are generated by SFWM, in which the signal photons are coupled out of the chip directly and used to generate the heralding signal locally. They are filtered out by an optical filter centered at the channel of C48 and detected by a superconducting nanowire single-photon detector (SNSPD, PHOTEC Inc.) with an efficiency of ~80%. The output of the SNSPD is loaded on a continuous wave (CW) laser light on the channel of C44 (Keysight N7714A) by an electro-optical intensity modulator, which is used as the optical heralding signal for the idler photons sent to the relay node. Its intensity is controlled by a variable optical attenuator to avoid noise photons generated by the optical heralding signal when it propagates in optical fibers. The idler photons of photon pairs are used to generate time-bin encoded single photon states on the chip, which shows a high fidelity of over 99% in the experiment. Details of the pulsed fiber laser system and the performance of the time-bin encoded single photon state generation are introduced in the Supplementary Informations S3 and S4. The idler photons with the time-bin encoded single-photon states and their optical heralding signals are multiplexed by a dense wavelength division multiplexer (DWDM) with a bandwidth of 200 GHz, and co-propagated over non-zero dispersion shifted fibers (G.655, Yangtze Optical Fibre and Cable) of 6.15 km to the relay node.

In the relay node, the quantum photonic circuit generates entangled photon pairs with time-bin encoded Bell states using the pulsed pump light from the same pulsed fiber laser system as the user node. The idler photons of the photon pairs are detected locally for the BSM, and the signal photons are sent to the central node over another spool of G.655 fibers of 6.15 km. The performance of entanglement distribution between the relay node and central node is demonstrated by Franson-type interference, which shows a fringe visibility of over 96% (excluding noise photons). Detailed results of the entanglement distribution are introduced in the supplementary information S5. In the relay node, the idler photons from the user node and their optical heralding signal pass through a variable optical delay line (ODL-1500PS, SC-Lightsource Inc.), then are separated by a DWDM. The optical heralding signal is pre-amplified by an erbium-doped fiber amplifier (EDFA) and detected by a high-speed photodetector (PD). The electrical signal of the PD is recorded by the time-correlated single photon counter (TCSPC, Timetagger Ultra, Swabian Instruments) at the relay node. The optical heralding signal is employed to herald the idler photons from the user node and is also utilized in the active feedback system for stabilization, as described in section “Materials and methods” and “The active feedback system for stabilization of the two-photon interference”. The idler photons from the user node pass through a polarization controller before they are coupled into the quantum photonic circuit of the relay node for BSM. The photons from the four output ports of the Bell state analyzer (C1–C4) are filtered by narrow-band optical filters at the channel of C40, respectively, and then detected by SNSPDs (their detection efficiency is about 90%). Their arrival times are recorded by the TCSPC. The states projected onto the Bell states $\left|{\Psi }^{+}\right\rangle$ and $\left|{\Psi }^{-}\right\rangle$ can be distinguished by analyzing the data recorded by the TCSPC, as detailed in section “Materials and methods” “Bell state measurement and HOM interference”. Besides, the photons output from the port C7 of the relay node are filtered by a narrow-band optical filter at the channel of C48, detected by an SNSPD, and recorded by the TCSPC at the relay node. It is also worth noting that the pulsed fiber laser system provides two electrical signals for the synchronization of TCSPCs at the relay node and the central node. One is sent to the TCSPC at the relay node directly, the other is converted to an optical synchronization signal by a CW laser and an electro-optical intensity modulator. The optical synchronization signal is combined with the idler photons generated in the quantum photonic circuit of the relay node by a DWDM, and then co-propagated to the central node over optical fibers of 6.15 km.

In the central node, a DWDM is used to separate the signal photons from the relay node and the optical synchronization signal. The optical synchronization signal is pre-amplified by an EDFA, detected by a PD, and recorded by the TCSPC (Timetagger Ultra, Swabian Instruments) at the central node for synchronization. The signal photons from the relay node are coupled into the quantum photonic circuit of the central node with the projective measurement system. The photons are output from the ports B1 and B2. Then they are filtered by narrow-band optical filters at the channel of C48 and detected by SNSPDs. The arrival times of these single-photon detection events are recorded by the TCSPC. The BSM results at the relay node and the projective measurement results at the central node are compared and analyzed, obtaining the four-fold coincidence counts to demonstrate the photonic quantum teleportation.

### Stability of two-photon interference

**Fig. 3:**
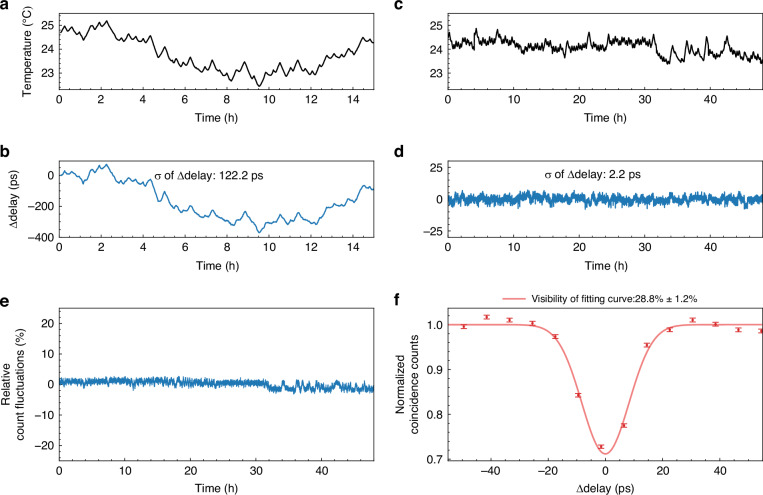
The performance of the active feedback system. **a**, **b** The room temperature and $\Delta_{delay}$ fluctuations without the stabilization by the feedback control. **c**, **d** Stabilized $\Delta_{delay}$ after activating the feedback system. *σ* represents the standard deviation of $\Delta_{delay}$ fluctuations. **e** Single-side count variation at one of the BSM output ports. **f** The normalized coincidence counts of the HOM interference experiment, with a fitted visibility of 28.8% ± 1.2%. Error bars arise from the Poisson distribution of photons

The implementation of the photonic quantum teleportation experiment depends on the performance of the BSM in the relay node. It requires that the photons from the user node and the idler photons generated locally in the relay node should be indistinguishable. However, the photons from the user node propagate along optical fibers of 6.15 km, and their arrival times would fluctuate due to the variation of ambient temperature. It would lead to an unstable arrival time difference, denoted as $\Delta_{delay}$, between the single-photon wavepackets from the user node and those from the relay node in BSM. An active feedback system with a variable optical delay line is used in the experiment to measure and stabilize $\Delta_{delay}$ (see sections “Materials and methods” and “The active feedback system for stabilization of the two-photon interference” for details). To show the effect of the active feedback system, the ambient temperature and $\Delta_{delay}$ are measured with and without the stabilization by the active feedback system. The results are shown in Fig. 3. Figure 3a, b show the results when the active feedback system is off. It can be seen that the fluctuation of $\Delta_{delay}$ is about 400 ps when the ambient temperature varies by 2 °C. The fluctuation is significantly exceeding the coherence time of the photons from the user node and the relay node, which is about 20 ps, determined by the bandwidth of the narrow-band optical filters used to select these photons. When the active feedback system is on, as shown in Fig. 3c, d, $\Delta_{delay}$ no longer depends on the ambient temperature and is highly suppressed with a standard deviation of ~2.2 ps over 48 h, far smaller than the photon coherence time. In addition, we have also monitored the single-side count variation at one of the BSM output ports on the relay node chip, as shown in Fig. 3e. The relative fluctuation of the single-side counts is approximately 3% over 48 h, indicating that the photon flux remains stable in the experimental system. It shows that the active feedback system can support stable quantum interference between the single-photon wavepackets from the user node and the relay node. It is demonstrated by the experiment of HOM interference between the photons from the user node and the idler photons generated locally, using the beam splitters in the Bell state analyzer on the chip. The upper limit of the HOM interference visibility ${V}_{{theory}}$ is given by the following equation^12,39^:

1 $${V}_{{theory}}=\frac{2}{{g}_{1}^{\left(2\right)}\left(0\right)\frac{{\bar{n}}_{1}}{{\bar{n}}_{2}}+{g}_{2}^{\left(2\right)}\left(0\right)\frac{{\bar{n}}_{2}}{{\bar{n}}_{1}}+2}$$

In the equation, *g*^(2)^(0) is the second-order correlation function at zero delay, and ${\bar{n}}_{1}$ and ${\bar{n}}_{2}$ represents the average photon numbers of photons from the user node and the relay node, respectively. To simplify the measurement in this experiment, we perform the HOM interference by two-fold coincidence measurements. The photons on both sides are in thermal states (${g}_{1}^{\left(2\right)}\left(0\right)={g}_{2}^{\left(2\right)}\left(0\right)=2$) when they are not heralded by their signal photons. The average photon numbers on both sides are equal, which is ensured by the results of photon counts at the output ports of the BSM (C1–4) in the measurement process. Hence, the fringe visibility of this interference has an upper limit of 33.3%. Figure 3f is the results of the normalized coincidence counts of the HOM interference experiment and its fitting fringe, showing a visibility (28.8% ± 1.2%) close to the upper limit. The stability of the two-photon interference ensures the success of the quantum teleportation experiment.

### Results of quantum teleportation

**Fig. 4:**
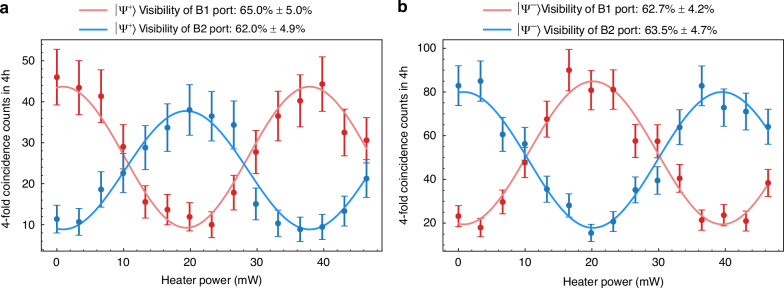
The experiment results of BSM when the single photon state sent by the user node is set to $|{\rm{\psi }}{\rm{\rangle }}=(|0{\rm{\rangle }}+{e}^{i{\phi }_{u}}|1{\rm{\rangle }})/\sqrt{2}$. **a**, **b** The 4-fold coincidence counts when the results of BSM are $\left|{\Psi }^{+}\right\rangle$ and $\left|{\Psi }^{-}\right\rangle$, respectively. Error bars arise from the Poisson distribution of photons

The performance of the chip-to-chip quantum teleportation is determined by the BSM at the relay node, which is tested first. In the measurement, the single photon state sent by the user node is set to $|{\rm{\psi }}\rangle$ $=(|0\rangle +{e}^{i{\phi }_{u}}|1\rangle )/\sqrt{2}$, corresponding to the equatorial state of the Bloch sphere. The phase ${\phi }_{u}$ could be adjusted by the TOPS at the VBS-UMZI in the quantum photonic circuit of the user node, shown in Fig. 1c. We fix the phases of the VBS-UMZIs at the relay node and central node (with the heater power of the TOPS set to zero), and only vary the phase ${\phi }_{u}$ at the user node. For each phase ${\phi }_{u}$, we record the photon arrival time data for 4 h from ports B1 and B2 of the VBS-UMZI at the central node. Since the Bell state analyzer is capable of distinguishing Bell states $\left|{\Psi }^{+}\right\rangle$ and $\left|{\Psi }^{-}\right\rangle$, we use the BSM results of $\left|{\Psi }^{+}\right\rangle$ to post-select the data from ports B1 and B2 (i.e., performing four-fold coincidence), which gives the interference fringes shown in Fig. 4a. Similarly, using BSM results $\left|{\Psi }^{-}\right\rangle$ for post-selection of the data from B1 and B2 yields the interference fringes shown in Fig. 4b. The blue and red dots are the four-fold coincidence counts when the photons at the central node output from ports B1 and B2, respectively, and the blue and red lines are their fitting curves by sinusoidal functions. It can be seen that all the results show sinusoidal interference fringes with varying ${\phi }_{u}$, confirming that the phase information of the single photon state sent by the user node is successfully transmitted to the central node by quantum teleportation over optical fibers of 12.3 km. The average visibility of these fringes is 63.3 ± 2.4%. The fidelity of the equatorial state can be estimated from the visibility of the interference fringes using ${F}_{{equator}}=(1+V)/2$ ^17,18^. Therefore, the corresponding average fidelity is 81.7%.

It is worth noting that since the success probability for distinguishing $\left|{\Psi }^{+}\right\rangle$ is 50% in the Bell state analyzer based on cascaded beam splitters, the maximum coincidence counts on the interference fringes related to $\left|{\Psi }^{+}\right\rangle$ are approximately half of those related to $\left|{\Psi }^{-}\right\rangle$, which are about 46 and 90, respectively. Hence, the rate of quantum teleportation events is about 34/h in this chip-to-chip quantum teleportation experiment over optical fibers of 12.3 km. Notably, at a heater power of 0 mW, all four interference fringes in Fig. 4 are at their extrema. Under these conditions, we define the phase ${\phi }_{u}=0$ at the user node and the phase ${\phi }_{c}=0$ at the central node. This allows us to define the relationship between input states and phase ${\phi }_{u}$, as well as the connection between measurement basis and phase ${\phi }_{c}$ at the central node. The detailed relationship between phase ${\phi }_{c}$, the output ports of the VBS-UMZI, and the specific measurement basis is discussed in “Materials and methods”.

Then, we prepare six input states ($\left|0\right\rangle$, $\left|1\right\rangle$, $\left|+\right\rangle$, $\left|-\right\rangle$, $\left|+i\right\rangle$, and $\left|-i\right\rangle$) at the user node and perform QST on the photons with the teleported state at the central node. The method of QST is discussed in “Materials and methods”. The measurement time is 24 h for each input state. When the result of BSM is $\left|{\Psi }^{+}\right\rangle$, the teleported state should be ${|{\psi }_{t}\rangle ={\boldsymbol{\sigma }}}_{x}|\psi \rangle$; when the result of BSM is $\left|{\Psi }^{-}\right\rangle$, the teleported state should be $|{\psi }_{t}\rangle ={{\boldsymbol{\sigma }}}_{y}|\psi \rangle$. The density matrices for the teleported states are reconstructed, and their state fidelities are calculated. The experiments of photonic quantum teleportation are performed over optical fibers of 12.3 km. It is also performed under a back-to-back case for comparison, in which the fiber spools are removed from the experimental system. The results are shown in Table 1. It can be seen that the fidelities of all the teleported states under fiber transmission exceed the classical limit of 2/3, with an average value of 81.2% when the result of BSM is $\left|{\Psi }^{+}\right\rangle$ and 81.0% when the result of BSM is $\left|{\Psi }^{-}\right\rangle$. It demonstrates that quantum teleportation is successfully implemented between the circuits of the three nodes over an optical fiber of 12.3 km in the experimental system. The detailed density matrices are provided in Supplementary Information S8.

**Table 1:** Comparison between fidelities of the quantum teleportation experiments obtained by QST over optical fibers of 12.3 km and those of the back-to-back case

Input state $|\psi \rangle$		$\left|0\right\rangle$	$\left|1\right\rangle$	$\left|+\right\rangle$	$\left|-\right\rangle$	$\left|+i\right\rangle$	$\left|-i\right\rangle$	Avg.
Teleported state		$\left|1\right\rangle$	$\left|0\right\rangle$	$\left|+\right\rangle$	$\left|-\right\rangle$	$\left|-i\right\rangle$	$\left|+i\right\rangle$	
BSM: $\left|{\Psi }^{+}\right\rangle$ ⇒ ${{\boldsymbol{\sigma }}}_{x}|\psi \rangle$	12.3 km	84.1%	86.4%	81.8%	77.0%	78.7%	79.3%	**81.2%**
	Back-to-back	88.3%	85.4%	80.8%	83.3%	78.9%	82.4%	**83.2%**
Teleported state		$\left|1\right\rangle$	$\left|0\right\rangle$	$\left|-\right\rangle$	$\left|+\right\rangle$	$\left|+i\right\rangle$	$\left|-i\right\rangle$	
BSM: $\left|{\Psi }^{-}\right\rangle$ ⇒ ${{\boldsymbol{\sigma }}}_{y}|\psi \rangle$	12.3 km	86.3%	87.6%	75.6%	79.0%	76.3%	80.9%	**81.0%**
	Back-to-back	93.1%	88.0%	83.6%	78.4%	80.1%	83.3%	**84.4%**

The bold values in Table 1 are specifically used to highlight the average values (Avg.), aiming to make the main results more prominent

## Discussion

Compared with the results of the back-to-back case, the average fidelity of the teleported states in the experiment with optical fibers decreases by 2% when the result of BSM is $\left|{\Psi }^{+}\right\rangle$ and by 3.4% when the result of BSM is $\left|{\Psi }^{-}\right\rangle$. It is mainly due to the impact of fiber dispersion on the photons from the user node when they transmit to the relay node over optical fibers of 6.15 km. The fiber dispersion would broaden the wavepacket of these photons, worsening the indistinguishability between the photons from the user node and the idler photons generated locally at the relay node when they are interfered in the Bell state analyzer in the quantum photonic circuit of the relay node. In this experiment, non-zero dispersion shifted fibers (G.655) are used, which have a dispersion parameter $D$ of 4 ps/(km·nm). The bandwidths of the optical filters for the idler photons are about 0.18 nm, leading to a temporal width ${T}_{0}$ of ~20 ps for the wavepacket of the photons in BSM. After fiber transmission of 6.15 km, the FWHM of the wavepacket, ${T}_{1}$, can be calculated using the following equation^40^:

2 $${T}_{1}={T}_{0}{[1+{(L/{L}_{D})}^{2}]}^{\frac{1}{2}}$$

where the dispersion length ${L}_{D}$ is defined as ${T}_{0}^{2}/|{\beta }_{2}|$, and ${\beta }_{2}$ is the group velocity dispersion, related to the fiber’s dispersion parameter $D$ by:

3 $${\beta }_{2}=-\frac{{\lambda }^{2}D}{2\pi c}$$

where $c$ is the speed of light. In the experimental setup, we calculate ${L}_{D}=78.5$ km, which is much larger than the fiber length used in the experiment. It can be expected that ${T}_{0}$ it would increase to 20.25 ps due to the fiber transmission. Besides, a frequency chirp would also be introduced in the wave packet due to the fiber dispersion. These effects would impact the fidelity of the teleported states. This impact could be avoided by using dispersion-shifted fibers or proper dispersion compensation to extend the fiber transmission distance.

It is worth noting that the fidelities of the teleported states in the back-to-back case also have spaces to be improved. They are mainly limited by the multi-pair events in the quantum light sources at the user node and the relay node, which impact the performance of BSM at the relay node. The performance could be improved if the pump lights in these quantum light sources are controlled at a lower level. Additionally, the temporal widths of the signal-photon wavepackets in the BSM could be broadened if optical filters with narrower bandwidths are used for selecting photons before detection. It is helpful to reduce the impact of residual fiber length fluctuations between the user node and the relay node on the quantum interference in the BSM, thereby increasing the fidelities of the teleported states. It can be expected that the rates of successful teleportation events would be reduced if the above two methods are used. Hence, quantum photonic circuits with smaller insertion losses are preferred to achieve these improvements.

A comparison of recent works on fiber-based quantum teleportation is shown in Table 2. Compared to previous works, in this work, most of the critical functions are integrated on chip, including the heralded single-photon generation, the entangled photon pair generation, the BSM, and the projective measurement. While achieving a miniaturized system, the teleportation rate and the fidelity of the teleported states are not as good as previous work using discrete devices. It is mainly due to the coupling loss between the fibers and the silicon photonic chip. Especially when four-fold coincidence is measured for teleportation, photons will pass through the fiber-chip interfaces multiple times, significantly reducing the system performance. To get an acceptable teleportation rate, relatively high pump power is used in the experiment, which induces more multi-pair events, consequently impacting the performance of BSM and the fidelity of the teleported states, consequently. It can be expected that the performance of fiber-chip coupling needs to be improved for better system performance. Besides, brighter quantum light sources with low-noise photon rates would also be helpful to overcome the additional losses. Thin film lithium niobate (TFLN) photonic chips with spontaneous parametric down conversion (SPDC) sources based on periodically poled lithium niobate (PPLN) waveguides are a way worth trying.

**Table 2:** A comparison of recent works on fiber-based photonic quantum teleportation. DOF: degree of freedom

Year	DOF	Quantum light source	Fiber type	Distance (km)	Fidelity	Rate (Hz)	Integration	Ref
2014	Time-bin	PPLN/PPKTP	Lab	25	81%	2 × 10^−3^	No	^42^
2015	Time-bin	DSF	Lab	102	84%	2 × 10^−2^	No	^8^
2016	Time-bin	DSF	Deployed	60	91%	5 × 10^−4^	No	^9^
2016	Time-bin	PPLN	Deployed	17	80%	2 × 10^−1^	No	^10^
2020	Path-polarization	Silicon ring	Lab	0.01	88.50%	***	Yes	^30^
2020	Time-bin	PPLN	Lab	44	89%	9 × 10^−3^	No	^11^
2022	Path-polarization	Silicon waveguide	Lab	0.01	89.40%	***	Yes	^31^
2023	Time-bin	PPLN	Deployed	64	91%	7.1	No	^12^
2024	Polarization	PPLN	Lab	30.2	89.90%	9 × 10^−2^	No	^13^
2025	Time-bin	Silicon waveguide	Lab	12.3	81%	1 × 10^−2^	Yes	Our work

As shown in Table 2, fiber-based quantum teleportation can also be realized by polarization-encoded states. In the two previous works on chip-to-chip quantum teleportation^30,31^, polarization-encoded states are used to achieve 10-meter fiber transmission. Recently, an experiment with 30.2-km fiber transmission was reported^13^, showing the potential of polarization-encoded states to realize long-distance fiber-based quantum teleportation. Its experimental setup is based on mature discrete fiber devices supporting low optical path losses. It achieves good performance on the quantum interference and BSM using quite bright sources based on SPDC in PPLN crystals. Compared to the time-bin encoding, the systems using polarization encoding with long-distance fiber transmission usually have higher requirements on polarization stability in fiber channels. In its experimental setup, the fiber spools for quantum teleportation are in the lab with thermal isolation, and the loose fibers are carefully fixed, ensuring the stability of fiber channels. It can be expected that active feedback control techniques should be applied if the system of teleportation is operated under deployed fibers.

## Materials and methods

### Projective measurement and QST

In the quantum photonic circuit of the central node, a VBS-UMZI is used to perform projective measurements on the time-bin encoded single photon state. When the VBS is set to a balanced state as a 50:50 beam splitter, the input state is projected onto three consecutive time bins (${t}_{0}$–${t}_{2}$) at the output ports of the VBS-UMZI, as shown in Fig. 1e. If the single photon at the central node is detected in time bins ${t}_{0}$ and ${t}_{2}$ in a four-photon coincidence event, the teleported state is projected to the measurement bases $\left|0\right\rangle,\,\left|1\right\rangle$, respectively. The four-fold coincidence counts of these two cases are denoted by ${n}_{0}$ and ${n}_{1}$. If the single photon at the central node is detected in a time bin ${t}_{1}$, which output port it is detected should be checked. The teleported state is projected to the measurement bases $\left|0\right\rangle +{e}^{i{\phi }_{c}}\left|1\right\rangle$ or $\left|0\right\rangle +{e}^{i\left({\phi }_{c}+\pi \right)}\left|1\right\rangle$, determined by the photon being detected at the port B1 or B2. Here, ${\phi }_{c}$ is the phase introduce by the VBS-UMZI at the central node. If ${\phi }_{c}$ is set to 0, the two measurement bases are $\left|+\right\rangle,\,\left|-\right\rangle$, respectively. The four-fold coincidence counts of these two cases are denoted by ${n}_{+}$ and ${n}_{-}$. If ${\phi }_{c}$ is set to $\pi /2$, the two measurement bases are $\left|+i\right\rangle,\,\left|-i\right\rangle$, respectively. The four-fold coincidence counts of these two cases are denoted by ${n}_{+i}$ and ${n}_{-i}$.

Based on the six projective measurement results, the density matrix of the teleported state $|{\psi }_{t}\rangle$, which is denoted by $\rho$, is reconstructed by QST. The density matrix is expressed as^9^:

4 $$\begin{array}{c}\rho =\frac{1}{2}\mathop{\sum }\limits_{i=0}^{3}{S}_{i}{\sigma }_{i}\\ {S}_{i}=\langle {\psi }_{t}|{\sigma }_{i}|{\psi }_{t}\rangle \\ {\sigma }_{0}=|0{{\rangle }}{{\langle }}0|+|1{{\rangle }}{{\langle }}1|=\left(\begin{array}{cc}1 & 0\\ 0 & 1\end{array}\right)\\ {\sigma }_{1}=|+{{\rangle }}{{\langle }}+|-|-{{\rangle }}{{\langle }}-|=\left(\begin{array}{cc}0 & 1\\ 1 & 0\end{array}\right)\\ {\sigma }_{2}=|+i{{\rangle }}{{\langle }}+{i|}-|-i{{\rangle }}{{\langle }}-{i|}=\left(\begin{array}{cc}0 & -i\\ i & 0\end{array}\right)\\ {\sigma }_{3}=|0{{\rangle }}{{\langle }}0|-|1{{\rangle }}{{\langle }}1|=\left(\begin{array}{cc}1 & 0\\ 0 & -1\end{array}\right)\end{array}$$

In the expression, ${S}_{i}$ represents the Stokes parameters. Due to the normalization, the value of ${S}_{0}$ is 1. In experiments, the Stokes parameters are related to the projective measurement results:

5 $$\begin{array}{l}{S}_{0} =\langle {\psi }_{t}|{\sigma }_{0}|{\psi }_{t}\rangle \\ \quad\,\,=\langle {\psi }_{t}|0\rangle \langle 0|{\psi }_{t}\rangle +\langle {\psi }_{t}|1\rangle \langle 1|{\psi }_{t}\rangle \\ \quad\,\,={P}_{|0\rangle }+{P}_{|1\rangle }=\frac{{n}_{0}+{n}_{1}}{{n}_{0}+{n}_{1}}=1\\ {S}_{1}={P}_{|+\rangle }-{P}_{|-\rangle }=\frac{{n}_{+}-{n}_{-}}{{n}_{0}+{n}_{1}}\\ {S}_{2} ={P}_{|+i\rangle }-{P}_{|-i\rangle }=\frac{{n}_{+i}-{n}_{-i}}{{n}_{0}+{n}_{1}}\\ {S}_{3} ={P}_{|0\rangle }-{P}_{|1\rangle }=\frac{{n}_{0}-{n}_{1}}{{n}_{0}+{n}_{1}}\end{array}$$

Here, ${P}_{|\psi \rangle }$ the probability of measuring the state $|\psi \rangle$.

In the experiment, two measurements are performed by setting the phase of the VBS-UMZI to 0 and $\pi /2$, each for 12 h. Thus, for a specific state sent from the user node, a measurement time of 24 h are required to obtain the experimental data for the density matrix reconstruction. The fidelities of the teleported states are calculated by^41^:

6 $$F({\rho }_{{aim}},\rho )={\left[{Tr}\left(\sqrt{\sqrt{{\rho }_{{aim}}}\rho \sqrt{{\rho }_{{aim}}}}\right)\right]}^{2}$$

Here, ${\rho }_{{aim}}$ is the density matrix of the teleported state, assuming that the quantum teleportation process is perfect.

### BSM and HOM interference

The quantum photonic circuit of the relay node uses a cascaded 50:50 beam splitter as the Bell state analyzer for the time-bin encoded two-photon states. The time-bin encoded Bell states, $|{\Psi }^{+}\rangle$ and $|{\Psi }^{-}\rangle$, can be distinguished by analyzing the coincidence counts of the single photon detection events at the four output ports of the Bell state analyzer (C1–C4) and two time-bins ${t}_{0}$ and ${t}_{1}$, as shown in Fig. 1d. If the two photons are in the state $|{\Psi }^{-}\rangle$, they would be separated by the quantum interference at the first beam splitter. Hence, one photon would be detected at the port C1 or C2, the other photon would be detected at the port C3 or C4, and they would be detected in different time bins. On the other hand, if the two photons are in the state $|{\Psi }^{+}\rangle$, they would output from the same port of the first beam splitter by the quantum interference, then be separated by the second beam splitter with a possibility of 50%. In this case, the two photons would be detected at the ports C1 and C2 (or C3 and C4), respectively, and they are also detected at different time bins. The two photons also have a possibility of 50% that they would be output from the same port at the two time bins. However, since the time difference of the two time bins is too small to be discriminated by the single photon detectors, this case could not be used to distinguish $|{\Psi }^{+}\rangle$ through coincidence measurement. If the two photons are in an unknown state, the above coincidence measurement results could be used to indicate whether the state is projected to $|{\Psi }^{-}\rangle$ or $|{\Psi }^{+}\rangle$. In the experiment, the BSM results are post-selected by the heralded signal from the user node through the three-fold coincidence measurement. The time tags of these single-photon detection events are stored and transmitted to the computer at the central node by local area network (LAN), in which they are compared with the projective measurement results of the teleported photons, completing the photonic quantum teleportation.

It can be seen that the quantum interference in the first beam splitter is crucial for the BSM. A HOM interference experiment is performed to test the performance of the quantum interference. In this experiment, the photon counting events from ports C1 and C2 are combined as the events from one output port of the first beam splitter using the channel merging function of the TCSPC, while the events from ports C3 and C4 are combined as those from the other output port. The coincidence counts between the two output ports of the first beam splitter are measured in the same time bin under different time difference $\Delta_{delay}$, performing the measurement of HOM interference.

### The active feedback system for stabilization of the two-photon interference

An active feedback system with a variable optical delay line is used in the experiment to measure and stabilize $\Delta_{delay}$, which is the arrival time difference in BSM between the single-photon wavepackets from the user node and those from the relay node. The optical heralding signal co-propagates with the time-bin encoded photons to the relay node, and their time delay after the optical fiber transmission would be adjusted simultaneously by a variable optical delay line. Then, the optical heralding signal is separated by a DWDM, amplified by an EDFA, detected by a PD, and recorded by the TCSPC at the relay node. We use the TCSPC to perform START–STOP measurements to determine the position of the optical heralding signal within each clock cycle, where the START signal is the clock synchronization signal from the pulsed fiber laser system, and the STOP signal is the optical heralding signal. The difference between the START signal and the STOP signal is denoted by ${T}_{{Herald}}$. Although the optical heralding signal is generated probabilistically since it reflects the single photon detection events of the signal photons at the user node, we can accumulate data over a long period (25 s in our experiment) to obtain an accurate ${T}_{{Herald}}$. Since the optical heralding signal and the photons from the user node propagate along the same fibers, ${T}_{{Herald}}$ it reflects the arrival time of the single-photon wavepacket from the user node with the impact of the length fluctuation of the transmission fibers. Besides, the photons output from the port C7 of the circuit of the relay node are also filtered, detected, and recorded by the TCSPC at the relay node. The same START–STOP measurements are performed, and here the difference between the START signal and STOP signal is denoted by ${T}_{L{ocal}}$, which indicates the timing reference of the idler photons generated locally in the relay node. By comparing ${T}_{{Herald}}$ and ${T}_{L{ocal}}$, $\Delta_{delay}$ can be calculated and used as a feedback signal to adjust the variable optical delay line. In this way, an active feedback control is applied to stabilize the quantum interference in BSM. During the experiment, the active feedback system adjusts the optical delay line every 25 s. It is worth noting that two indoor fiber spools are used as the transmission fiber in this experiment. The polarization state of the photons is well preserved when they propagate through the optical fibers in the lab environment. Hence, no active feedback method is applied to stabilize their polarization.

## Supplementary information

Supplementary Information for Chip-to-chip photonic quantum teleportation over optical fibers of 12.3 km

## Data Availability

All data needed to evaluate the conclusions in the paper are present in the paper and/or the Supplementary Information. Additional data related to this paper may be requested from the authors.
