# Supplementary material for: Chip-to-chip photonic quantum teleportation over optical fibers of 12.3 km
Source: Light Sci Appl. 2025 Jul 9;14:243. doi: 10.1038/s41377-025-01920-z (PMC12241450; doi:10.1038/s41377-025-01920-z)
Supplement: Supplementary file 1 — Supplementary Information for Chip-to-chip photonic quantum teleportation over optical fibers of 12.3 km [file 41377_2025_1920_MOESM1_ESM.pdf]

Supplementary Information for

Chip-to-chip photonic quantum teleportation over optical fibers of 12.3 km

Dongning Liu,[1] Zhanping Jin,[1] Jingyuan Liu,[1] Xiaotong Zou,[1] Xiaosong Ren,[1] Hao Li,[2] Lixing You,[2] Xue Feng,[1] Fang Liu,[1] Kaiyu Cui,[1] Yidong Huang,[1,3*] and Wei Zhang,[1,3*]

[1] Frontier Science Center for Quantum Information, Beijing National Research Center for Information Science and Technology (BNRist), Electronic Engineering Department, Tsinghua University, Beijing 100084, China.

[2] National Key Laboratory of Materials for Integrated Circuits, Shanghai Institute of Microsystem and Information Technology, Chinese Academy of Sciences, Shanghai 200050, China.

[3] Beijing Academy of Quantum Information Sciences, Beijing 100193, China.

** Corresponding author. E-mail address: yidonghuang@tsinghua.edu.cn (Y.H.) and zwei@tsinghua.edu.cn (W.Z.).*

S1. The performances of on-chip devices

S1.1 VBS-UMZIs and the delay waveguides

We use an optical sweep system (Santec TSL 570 and MPM 210H) to measure the transmission spectra of the packaged chip. A current source (T2-MS64-501CCR, Time-transfer Optoelectronics co., ltd.) is used to realize on-chip phase control by the thermo-optic phase shifters (TOPSs). Fig. S1 shows the transmission spectra of the VBS-UMZI on the quantum photonic circuit of the central node near the central frequency of ITU channel of C40, including the losses introduced by the two edge couplers. The relative intensity of the lights traveling through the short and long arms is controlled by the TOPS of the VBS.

Fig. S1a and c show the transmission spectra when the light entirely passes through the short and long arms, respectively. Their transmission difference is about 0.65 dB, calculated by averaging the transmission losses over the spectra of them. Since the length difference of the two arms is 3.2 cm, the propagation loss of the rib waveguide is about 0.2 dB cm-1.

Fig. S1b shows the transmission spectrum when the VBS is used to balance the two outputs of the VBS-UMZI. In this condition, the light propagates through the short and long arms simultaneously and interferes at the last beam-splitter, leading to an obvious sinusoidal fringe with an extinction of about 20 dB. Fig. S1d presents the detailed transmission spectrum in this condition, showing that the period of the interference fringe is about 0.02 nm (2.5 GHz). It agrees with the design that the arm difference of the VBS-UMZI is 400 ps.

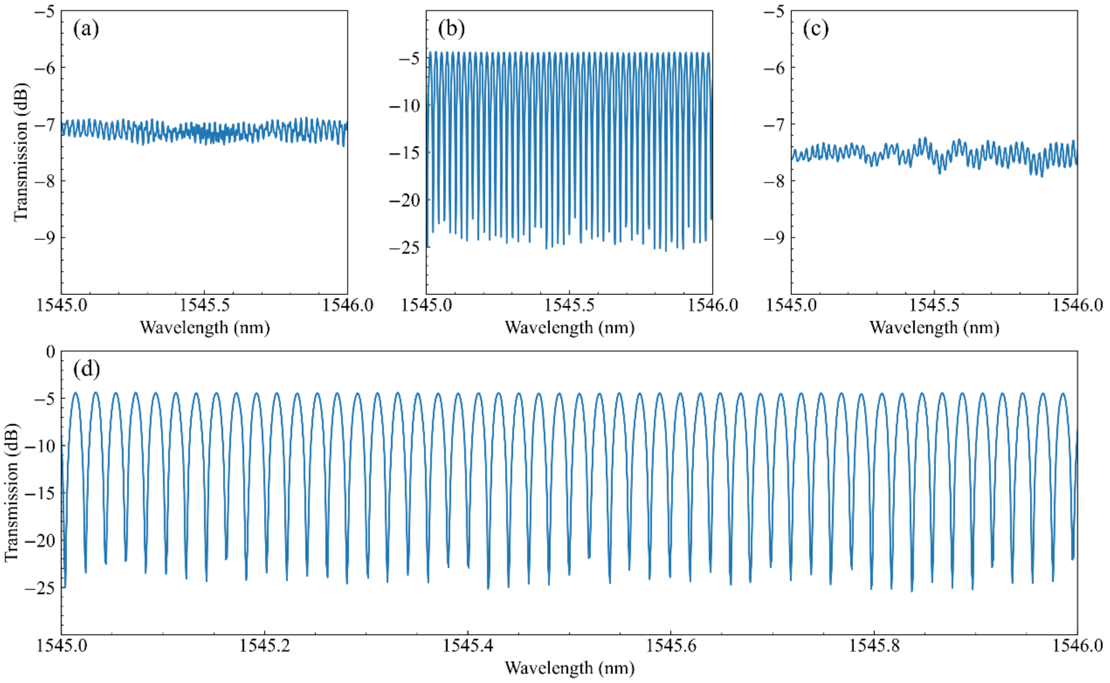

Fig. S1. (a) and (c) The transmission spectra when the light propagates through the short and long arms of the VBS-UMZI, respectively. (b) The spectrum when the VBS is set to balance the two outputs of the VBS-UMZI. (d) Detail spectrum showing the period of the interference fringe.

To estimate the phase stability of the VBS-UMZI in response to temperature fluctuations, we calculated the expected phase shift for a VBS-UMZI using the thermal expansion coefficient and the thermo-optic coefficient of silicon. The phase shift $\Delta\phi$ is given by the equation:

$$\begin{aligned}\Delta\phi &= \phi_{(T+\Delta T)} - \phi_{(T)} \\ &= \frac{2\pi}{\lambda}(L + \alpha L\Delta T)(T + \Delta T)\frac{\mathrm{d}n}{\mathrm{d}T} - \frac{2\pi}{\lambda}Ln_{eff}\end{aligned} \quad \text{(S1)}$$

where λ is the central wavelength (1542 nm), L is the length difference between two arms (3.2 cm), α is the thermal expansion coefficient of silicon (2.57e-6 at room temperature[1]), $\frac{\mathrm{d}n}{\mathrm{d}T}$ is the thermo-optic coefficient of silicon (1.8e-4 at room temperature[2]), and ΔT is the temperature change. Assuming the temperature change is ±0.002 K (as provided by the stability of the TEC controller), we calculate the resulting phase shift to be approximately 5.5°. This corresponds to a phase error of about 1.5%, which is considered an acceptable error for our chip-based experimental system. Given these considerations, we are confident that the TEC systems, along with the estimated phase stability, will provide sufficient temperature stability for the experiment of quantum teleportation. Above analysis is also valid when the three quantum photonic circuits are on three chips and their

temperatures are controlled separately at different locations in scenarios of field-based implementations.

S1.2 Waveguide Bragg grating band-stop filters

The quantum photonic circuits of the user node and the relay node use waveguide Bragg grating (WBG) band-stop filters[3–6] to suppress the pump light after the single mode silicon waveguides for spontaneous four-wave mixing (SFWM). The WBG consists of a piece of waveguide and the periodic corrugations on the sidewall. Specifically, the waveguide should be wide enough to support higher order modes, the corrugation act as Bragg gratings to enable the phase matching condition between the forward propagating fundamental mode (TE0) and the backward propagating higher order mode (TE1), and then the higher order mode would be dissipated in the taper and the bent waveguide before the WBG. At the wavelength where phase matching condition is met, the WBG would cause the reflection and dissipation of the light, which essentially act as a band-stop filter for pump light[7–9]. Fig. S2a is the sketch of the device. The width of the waveguide (W_{wg}) is 1 μm, with the gratings staggered on both sides of the waveguide. The width (W), period (P) and duty cycle of the gratings are 150 nm, 293 nm and 50%, respectively. Fig. S2b shows the transmission spectrum of a WBG band-stop filter on the chip (excluding the coupling loss). It can be seen that it supports a stop band with a central wavelength of 1540.5 nm and a 3 dB stop-band width of 4.2 nm. The extinction ratio of the stopband exceeds 40 dB. There are small deviations on the central wavelengths of different WBG band-stop filters on the chip (1~2 nm) due to the process deviation in chip fabrication, which can be adjusted by the TOPSs on WBGs.

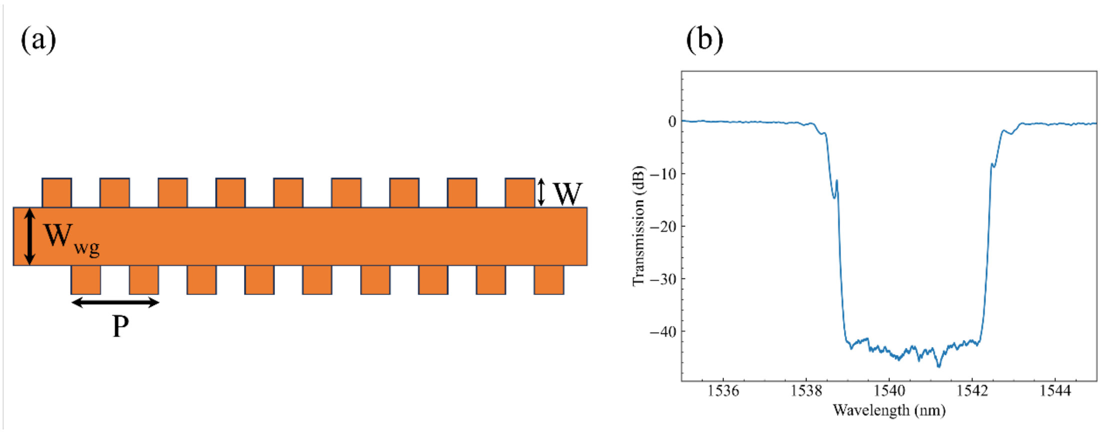

Fig. S2. Waveguide Bragg grating band-stop filters (a) the sketch, (b) the measured transmission

spectrum.

S2. Transmission spectra and minimum insertion losses of typical optical paths on the chip

To show the performance of the chip, we measure transmission spectra of some typical optical paths and show them in Fig. S3. In the measurement, the temperature of the chip is stabilized at 15 °C and the stop band of the WBG filters are adjusted to cover the wavelength of the pump light. The TOPSs of the VBSs and the 1600 GHz-UMZIs are also adjusted to match the conditions of the quantum teleportation experiment. Fig. S3a and b show the results when the light injects into the quantum photonic circuit of the user node from the port A_{in} and output from the ports A1 and A3, which are output ports for the heralding photons and the idler photons sent to the relay node, respectively. Fig. S3c and d show the results when the light injects into the quantum photonic circuit of the relay node and output from the ports C1 and C6, which are output ports for the photons participating in BSM and signal photons sent to the central node, respectively. In the experiment of photonic quantum teleportation, the wavelengths of the pump light, the signal photons and idler photons are set at 1542.14 nm (ITU C44), 1538.98 nm (ITU C48) and 1545.32 nm (ITU C40), respectively. The green-, blue- and red-shaded regions indicate the bands of optical filters for them. It can be seen that in the spectra shown in Fig. S3b and c, which related to the idler photons generated in the user node and the relay node, the transmission losses at the channel of C40 are much smaller than those at the channel of C48. On the other hand, in the spectra shown in Fig. S3a and d, which related to the signal photons generated in the user node and the relay node, the transmission losses at the channel of C48 are much smaller than those at the channel of C40. These results show the effects of 1600GHz-UMZIs on separating the signal photons and idler photons. It also can be seen that in all the spectra the transmission losses at the channel of C44 is high, showing the effects of the WBG band-stop filters to suppress the pump light. It is worth noting that in the measurements of the spectra shown in Fig. S3b~d, the lights pass through VBS-UMZIs with a large arm difference of 400 ps. Hence, dense interference fringes can be observed in these spectra. The results shown in Fig. S3 demonstrate that all the optical filters on the chip operate as the designs of these quantum photonic circuits.

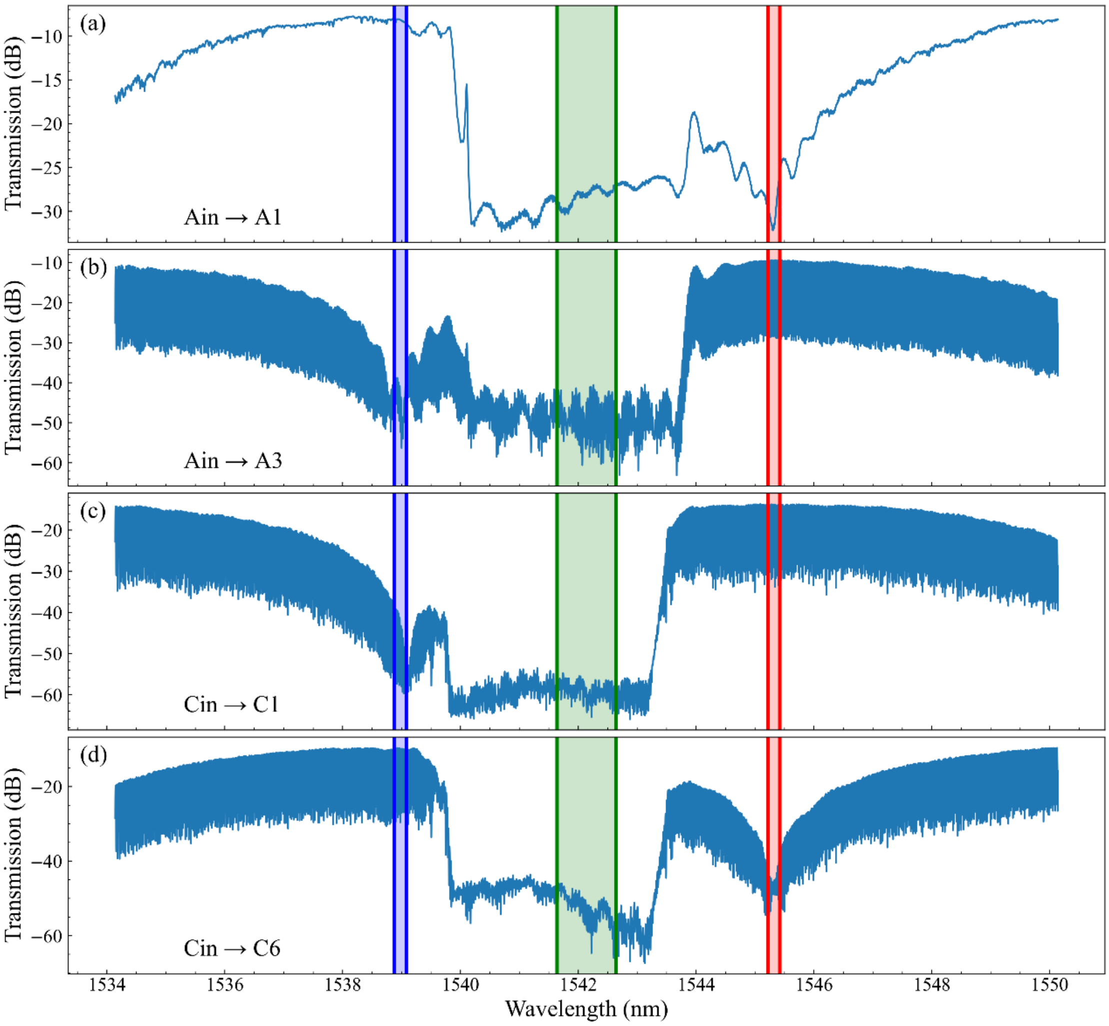

Fig. S3. Transmission spectra of some input-output ports on the chip. (a) The output port of heralding photons at the user node (A_{in}→A1), (b) the output port of idler photons sent to relay node (A_{in}→A3), (c) the output port of BSM at the relay node (C_{in}→C1), and (d) the output port of signal photons sent to the central node (C_{in}→C6). The green-shaded region represents the wavelength range of pump light, and the blue and red regions indicate the optical filtering bands of the signal and idler photons at the channels of C48 and C40, respectively.

The minimum insertion losses of some typical optical paths on the chip (including the input and output edge couplers) are summarized in Table S1. The fiber-to-chip coupling loss is about 2 dB per facet. It can be seen that the insertion losses from the port B_{in} to the ports B1 and B2 are 5 dB, in which 4 dB is due to the fiber-chip coupling at the two sides and only 1 dB is contributed to the on-chip loss of the VBS-UMZI in the quantum photonic circuit at the central node. It shows the effect of the low-loss delay waveguide in the long arm of the VBS-UMZI. The insertion losses of the paths C_{in}→C1~4 and C5→C1~4 are over 10 dB, which have the splitting losses of 6 dB due to cascaded beam-splitters for BSM.

Table S1. Minimum insertion losses of some typical optical paths on the chip

Input port → Output port	Output photons of the optical paths	Insertion Loss (dB)
A_{in} → A1	Heralding photons at the user node	7.5
A_{in} → A2~3	Idler photons sent to the relay node	9.5
C_{in} → C1~4	Photons participate in BSM at the relay node	14.5
C_{in} → C6	Signal photons sent to the central node	9
C5 → C1~4	Photons participate in BSM at the relay node	11
B_{in} → B1~2	Photons after the projective measurements at the central node	5

S3. Pulsed fiber laser system and optical filters for the pump light, signal photons and idler photons

The pulsed fiber laser system in the experimental setup is shown in Fig. S4, which provides pulsed pump lights at 1542.14 nm (ITU C44) for both the user node and the relay node. In the system, a mode-locked fiber laser generates femtosecond laser pulses periodically with a repetition rate of 100 MHz. The pulsed light pass through a DWDM, which performs as an optical filter at the channel of ITU-C44. Then it is amplified by an EDFA. The amplified light pass through cascaded optical filters (including a band-pass optical filter with a bandwidth of 40 nm and a cascaded DWDM) to remove the noise photons such as amplified spontaneous emission (ASE) of the EDFA. After that, it is split into two paths by a 50:50 fiber coupler to the user node and relay node, respectively. In each path, a cascaded DWDM is used to further remove the noise photons and a polarization controller is used to adjust the polarization state of the pulsed pump light when it is coupled to the chip. The output power of the pulsed pump light is stabilized by a power stabilization unit, which includes a variable optical attenuator (VOA, Keysight 81570A), a 99:1 fiber coupler, and an optical power meter (Keysight N7744A). A small part of the light is coupled to the optical power meter by the fiber coupler. A PID algorithm is applied to control the VOA according to the measured power of the optical power meter. The time delay difference between the two paths after the 50:50 fiber coupler is greater than 10 ns, ensuring that the quantum light sources in the two nodes are pumped by different light pulses.

Besides, the mode locked fiber laser also provides an electrical signal synchronized with its output optical pulses. It is separated into two SYNC signals by a digital delay/pulse generator (DG645,

Stanford Research Systems). One is used to synchronize the two TCSPCs at the relay node and the central node. The other is used as the START signal mentioned in Materials and methods 4.3 in the manuscript.

It is worth noting that in a real-world experiment, two pulsed lasers are required for the user and relay nodes. The pulsed pump lights for both nodes need to be synchronized using techniques such as phase-locked loops. Previous works have shown that, with the use of active synchronization, the time jitter between the pulses generated from two lasers can be reduced to 100 fs[10], which meets the experimental requirements for quantum teleportation. Besides, weak pulsed lights, which propagate along the same fibers with the single photon wavepackets, are also commonly used as synchronization signals to support such experiments[11–14].

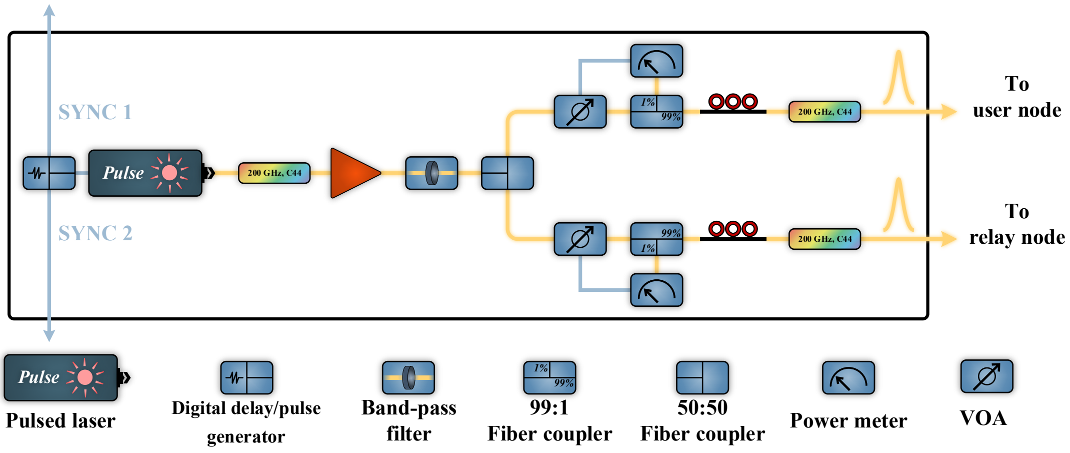

Fig. S4. The pulsed fiber laser system. The system includes a mode-locked fiber laser, an EDFA, a series of optical filters, a polarization controller, a 50:50 fiber coupler and two power stabilization units each with a VOA, a 99:1 fiber coupler, and a power meter.

In the experiment, the filters for the pulsed pump light have a 3 dB bandwidth of ~1 nm, and an insertion loss of ~0.8 dB. The filters for the signal and idler photons have a 3 dB bandwidth of ~0.18 nm, and an insertion loss of ~2 dB. The transmission spectra of these filters are measured and shown in Fig. S5. It can be observed that the spectra of filters with the same central wavelength are nearly identical. Estimated by the time-bandwidth relation of a Gaussian pulse, the temporal width of the signal and idler photons is about 20 ps, and that of the pump pulses is about 3.5 ps. The large difference between the optical filter bandwidths of the pulsed pump light and idler photons participating in BSM ensures a good indistinguishability between the photons sent by the user node

and generated locally at the relay node, which is an important condition to achieve high-quality BSM in the experiment[15,16].

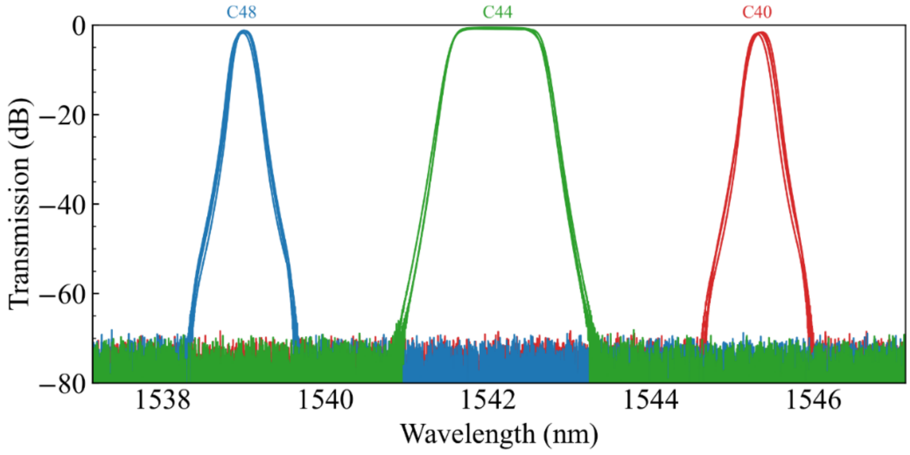

Fig. S5. Transmission spectra of optical filters for the pulsed pump light, signal photons and idler photons.

S4. Time-bin encoded single-photon state generation

The quantum photonic circuit of the user node is designed to generate heralded single photons with time-bin encoded states, which are demonstrated experimentally. The experimental setup shown in Fig. S6a is used to show the performance of single photon state generation when the state locates at the two poles of the Bloch sphere ($|0\rangle$ and $|1\rangle$). Pulsed pump light is injected into the quantum photonic circuit at the user node and photon pairs are generated by SFWM. The signal photons are detected directly to produce the heralding signal. The idler photons are encoded using the VBS-UMZI. By adjusting the VBS to fully manipulate the idler photons to the early time-bin t_0 and the late time-bin t_1, the state of idler photons is encoded as $|0\rangle$ and $|1\rangle$. The time-resolved coincidence counts (back-to-back case without fiber transmission) of the photon pairs are measured when the state of the idler photons is $|0\rangle$ and $|1\rangle$ and the results are shown in Fig. S6b and c. Since the photons in $|0\rangle$ and $|1\rangle$ passes through the short arm and long arm of the VBS-UMZI, respectively, and the loss of the long arm is a little higher than the loss of the short arm, the coincidence counts shown in Fig. S6c is slightly lower than that in Fig. S6b. By properly adjusting the VBS, the photons also can be distributed to the time bins t_0 and t_1 equally. The time-resolved coincidence counts in this case are also measured and shown in Fig. S6d. The time difference of the two coincidence peaks can be calculated by Gaussian fitting, which is 401.6 ± 1.0 ps, matching the design value of 400 ps. Fig. S6e and f shows the heights of the two coincidence peaks at time bins

t_0 and t_1 under different heater power on the TOPS of the VBS under back-to-back case and 6.15 km fiber transmission case, respectively. The heater power, which is proportional to the phase of TOPS, is calculated by the current applied on the TOPS and the resistance of TOPS (~3 kΩ). The average visibility of two sinusoidal interference fringes under back-to-back case is 98.55%, and those under 6.15 km fiber transmission case is 98.60%. Similarly, due to the slightly higher loss in the longer arm of the VBS-UMZI, the interference fringe corresponding to time-bin t_1 has a lower peak than that of time-bin t_0. On the other hand, the noise counts at both time bins are the same, which results in the visibility at t_1 being slightly lower than that at t_0.

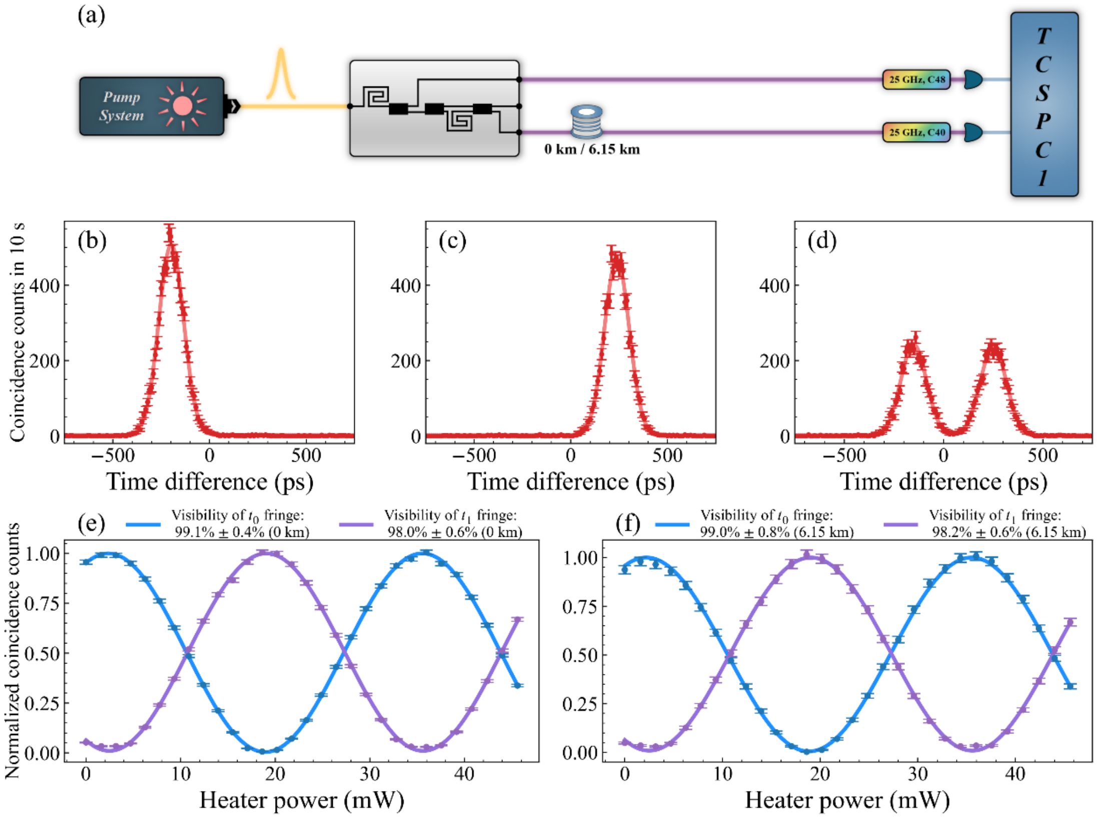

Fig. S6 The performance of single photon state generation when the state locates at the two poles of the Bloch sphere. (a) Experimental setup. (b) and (c) The time-resolved coincidence counts when the state of the idler photons is $|0\rangle$ and $|1\rangle$. (d) The time-resolved coincidence counts when the photons distribute to the time bins t_0 and t_1 equally. (e) and (f) Heights of the two coincidence peaks at time bins t_0 and t_1 under different heater power on the TOPS of the VBS, under back-to-back case and 6.15 km fiber transmission case, respectively. Error bars arise from the Poisson distribution of photons.

When VBS has been set that the idler photons are distributed to the time bins t_0 and t_1 equally. The state of the photons can be encoded into four typical states at the equator of the Bloch

sphere ($|+\rangle$、$|-\rangle$、$|+i\rangle$、$|-i\rangle$). The performance of the heralded single photon source at the user node when it is set to these states is demonstrated by the setup shown in Fig. S7a. The idler photons are sent to the quantum photonic circuit at the central node, which perform the projective measurement on the time-bin encoded state. The time-resolved coincidence counts between the signal photons detected for the heralding signal and the idler photons after the projective measurement are measured with varying the phase α of the VBS-UMZI at the circuit of the central node. When the state of idler photons is encoded in $|+\rangle$, the measurement results under $\alpha = 0$ and $\alpha = \pi$ are shown in Fig. S7b and c, respectively. It can be seen that there are three coincidence peaks in the results. The central peak shows the interference between the wavepackets in the time bins t_0 and t_1, which can be used to demonstrates that idler photons is encoded in $|+\rangle$. Fig. S7d and e show the variation of the height of the central peak under different α, under back-to-back case and 6.15 km fiber transmission case, respectively. The results show two sinusoidal fringes with the visibility V, which is over 99%. The results shown in Fig. S7b and c correspond to the maximum and minimum of the fringe, respectively. The fidelity of the generated single photon state (denoted by F) , can be calculated by[17,18]:

$$F = \frac{1+V}{2} \tag{S2}$$

which is higher than 99%. Other states at the equator of the Bloch sphere also demonstrated in this way and the results are similar with the case of $|+\rangle$.

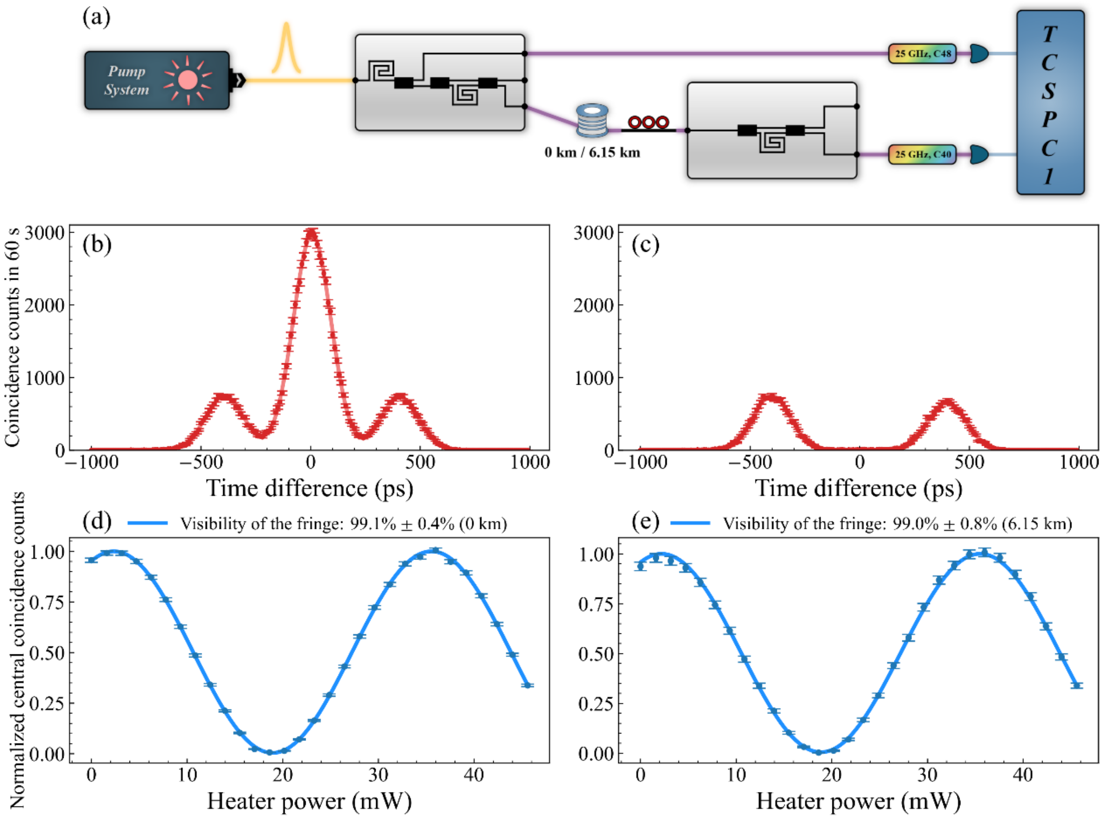

Fig. S7 The performance of single photon state generation when the state locates at the equator of the Bloch sphere. (a) Experimental setup. (b) and (c) The time-resolved coincidence counts under $\alpha = 0$ and $\alpha = \pi$ when the state of idler photons is encoded in $|+\rangle$. (d) and (e) The normalized height of the central coincidence peak under different α, under back-to-back case and 6.15 km fiber transmission case, respectively. Error bars arise from the Poisson distribution of photons.

These results demonstrate that the time-bin encoded single-photon states generated by the user node maintain high quality even after transmission through 6.15 km of optical fiber.

S5. Experiment of Franson-type interference

The experiment of photonic quantum teleportation relies on high-quality distribution of the time-bin encoded entangled state between the relay node and the central node. It is demonstrated by an experiment of the Franson-type interference[19,20], and the experimental setup is shown in Fig. S8a. At the relay node, one output port of the Bell state analyzer is selected to extract the idler photons generated locally in the relay node. They pass through an off-chip UMZI (Kylia MINT) with an arm difference of 400 ps, then detected by a SNSPD after optical filtering and recorded by the TCSPC at the relay node. The signal photons generated at the relay node are sent to the central node over optical fibers of 6.15 km. The VBS-UMZI in the quantum photonic circuit of the central node is

used as another UMZI with an arm different of 400 ps for the signal photons. The photons pass through the circuit of the central node and then detected by a SNSPD after optical filtering and recorded by the TCSPC at the central node. The two TCSPCs at the relay node and the central node are synchronized by the same method with that in the experiment of photonic quantum teleportation.

The phase of the off-chip UMZI at the relay node is denoted by β, which is set to $\beta = \varphi$ and $\beta = \varphi + \pi/2$ in the experiment. Meanwhile, the phase α of the VBS-UMZI at the quantum photonic circuit at the central node is scanned by controlling the TOPS on its short arm. Photons output from VBS-UMZI are located in three constructive time bins. Single photon events recorded in the central time bin are post-selected by the synchronization signal. Two-fold time-resolved coincidence measurements are then performed between these post-selected events and the single photon events recorded at the relay node for Franson-type interference[20]. The measurement time is 30 minutes under a specific combination of β and α. The height of the central peak of the time-resolved coincidence counts is recorded to obtain fringes of the Franson-type interference. The measurement results are shown in Fig. S8b, where the red and blue dots are normalized height of the central peak under $\beta = \varphi$ and $\beta = \varphi + \pi/2$ after subtracting accidental coincidence counts, respectively. The red and blue lines are their sinusoidal fitting curves with net visibilities of 98.8% ± 5.4% and 96.1% ± 4.3%, respectively. Their raw visibilities are 87.4% ± 5.1% and 85.0% ± 3.8%. Both visibilities exceed the threshold of 70.7%. These results demonstrate that the experimental system supports the distribution of time-bin encoded entangled states between the relay node and the central node over optical fibers of 6.15 km.

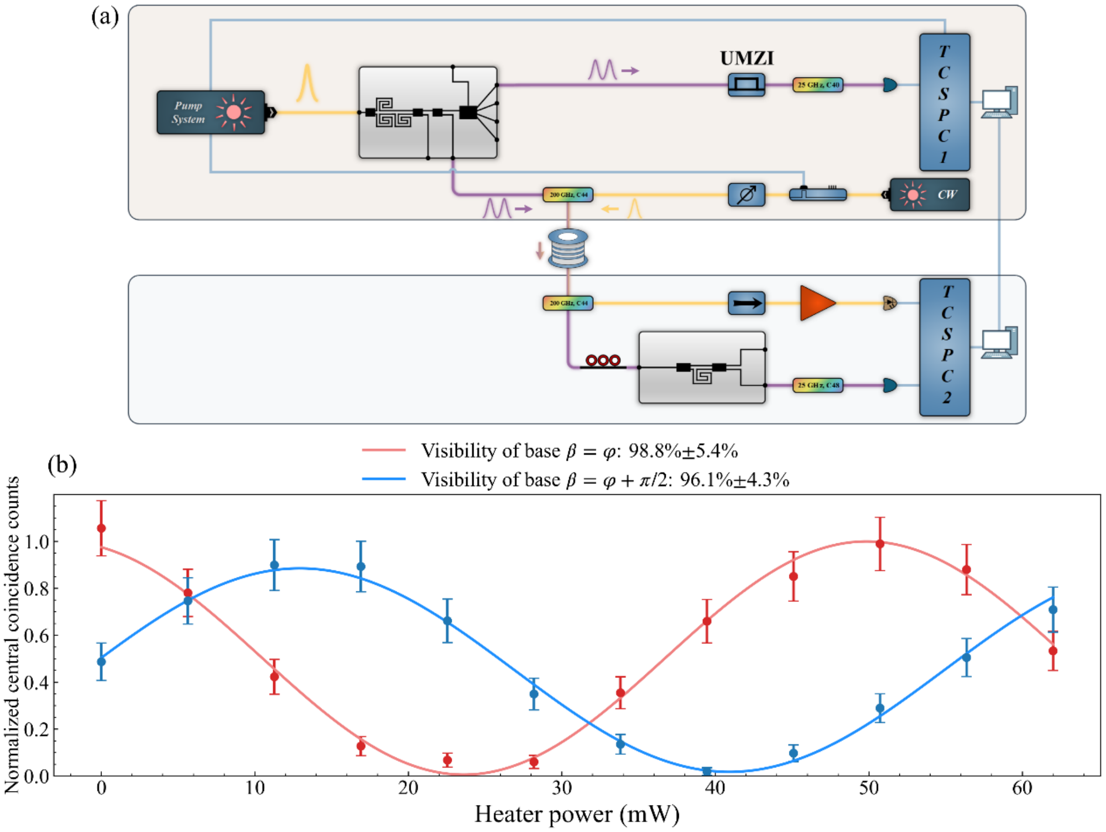

Fig. S8 The Franson-type interference experiment to demonstrate the entanglement distribution between the relay node and the central node. (a) The experimental setup. (b) Normalized interference fringes after subtracting the accidental coincidence counts under two measurement bases, with net visibilities of 98.8% ± 5.4% and 96.1% ± 4.3%. Error bars arise from the Poisson distribution of photons.

S6. Experimental results under back-to-back case

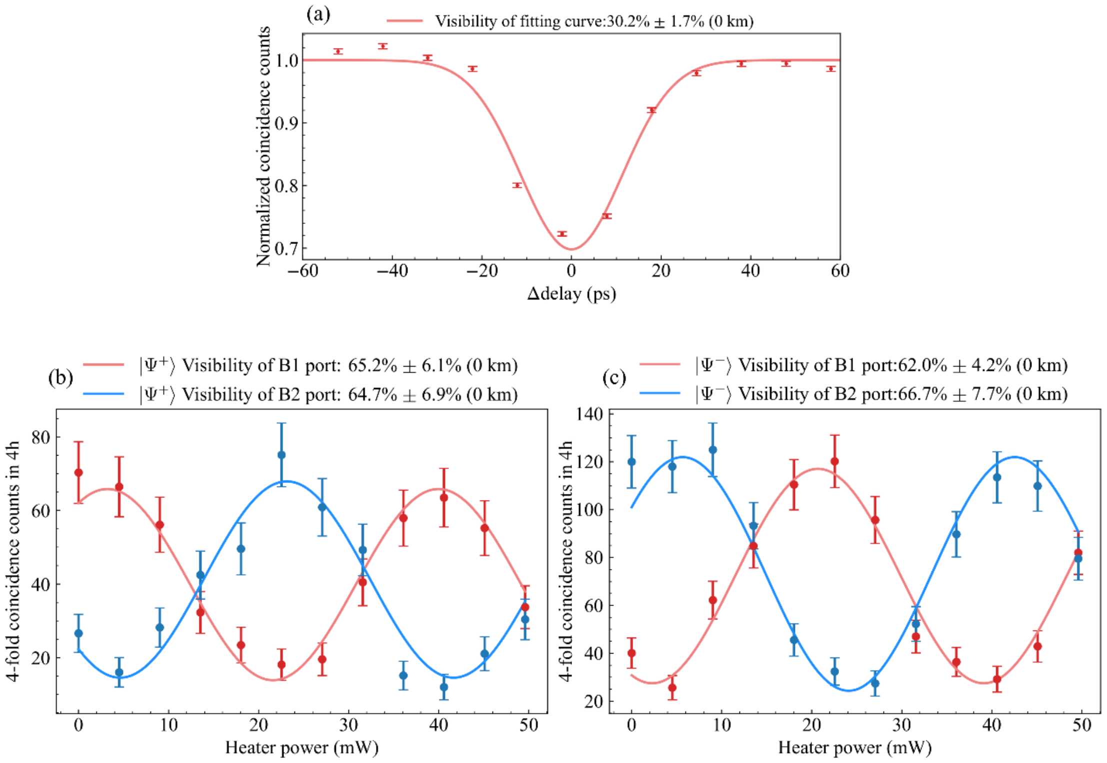

Fig. S9 (a) The normalized coincidence counts of the HOM interference experiment under back-to-back case, with a fitted visibility of 30.2% ± 1.7%. (b) and (c) The 4-fold coincidence counts under back-to-back case when the results of BSM are $|\Psi^{+}\rangle$ and $|\Psi^{-}\rangle$ respectively. The average visibility of four fringes is 64.7%. Error bars arise from the Poisson distribution of photons.

S7. The heralding efficiency of user node

We take an experiment to measure the heralding efficiency of the quantum light source at the photonic circuit of the user node under the same pump condition with the quantum teleportation experiment. The experiment setup is shown in Fig. S6a in the supplementary. To measure the heralding efficiency, the fiber spool of 6.15 km is not required. The heralding efficiency is indicated by the ratio of coincidence counts of the signal and idler photons to the single-side counts of the signal photons, which is measured to be about -15.5 dB.

It is worth noting that the on-chip loss (including the fiber-chip coupling loss) of the idler photons could be estimated through the measured insertion losses shown in Table. S1. It can be seen that the insertion loss of the circuit of the user node between the input port and the ports of idler photons (Ain → A2~3) is 9.5 dB. Considering that the fiber-chip coupling at the input port is not related to the idler photons (reducing 2 dB), and no interference occurs at the last 50:50 beam splitter

in the VBS-UMZI since the temporal width of the idler photon wavepacket is quite short (increasing 3 dB), the on-chip loss of the idler photons is about 10.5 dB. Besides, the narrow band filter for the idler photons in the setup have an insertion loss of 2 dB, and the efficiency of the superconducting nanowire single-photon detector (SNSPD) is about 80% (1 dB). The theoretical value of the coincidence to single side count ratio is about - 13.5 dB. The deviation of the measured result to the theoretical value is due to the impacts of noise counts, especial the noise photons generated by spontaneous Raman scattering when the pump light propagates along optical fibers before it injects into the chip. As pointed out by Ref. 21, a piece of 20-cm fiber will induce noise counts with a rate up to dozens of kcps[21]. The fiber length before the chip in our setup is about 2 m, it can be expected that this effect would greatly impact the coincidence to single side count ratio, i. e., the heralding efficiency.

S8. Reconstructed density matrices of the teleported states

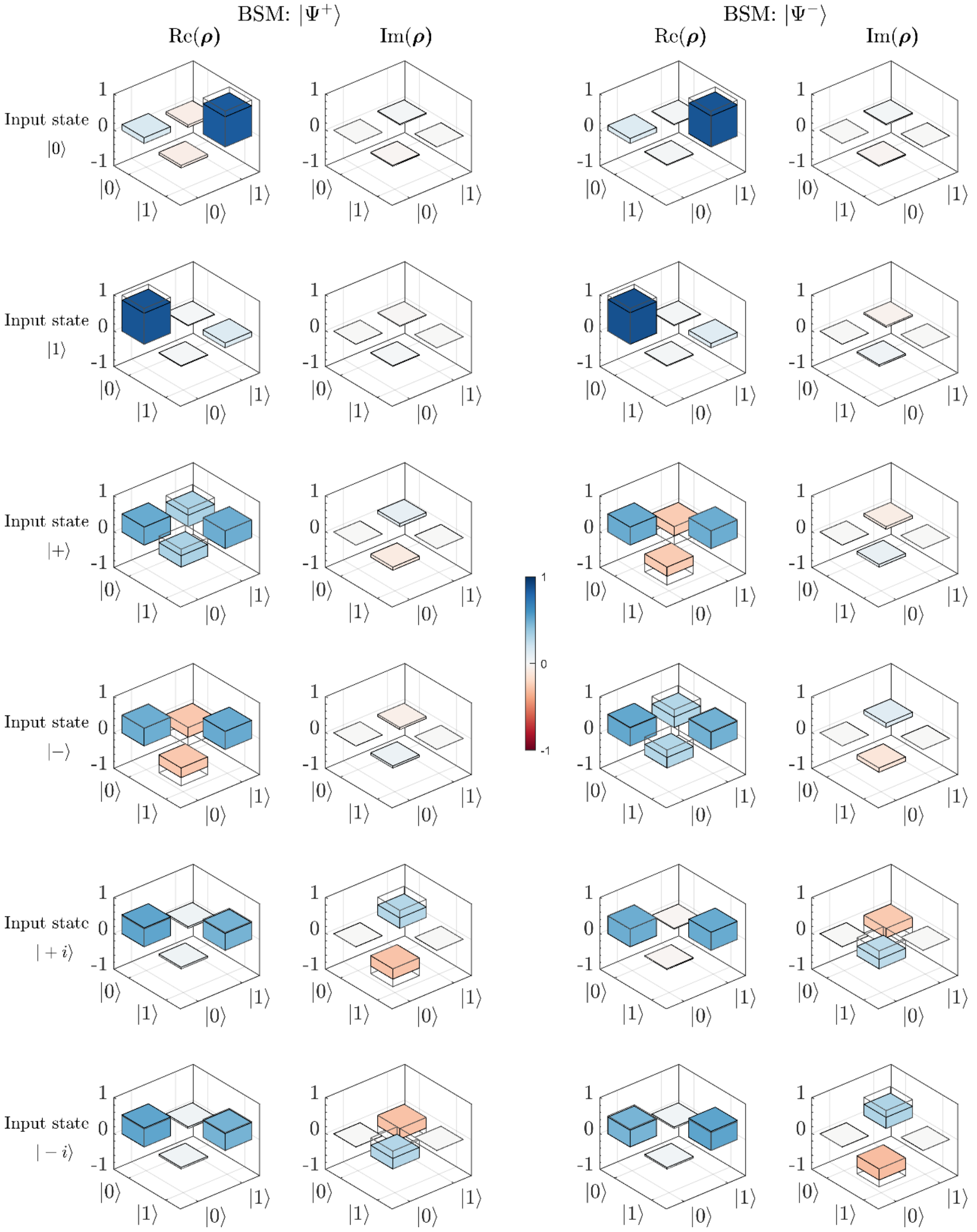

Fig. S10 Reconstructed density matrices of the teleported states in the experiment with optical fiber transmission.

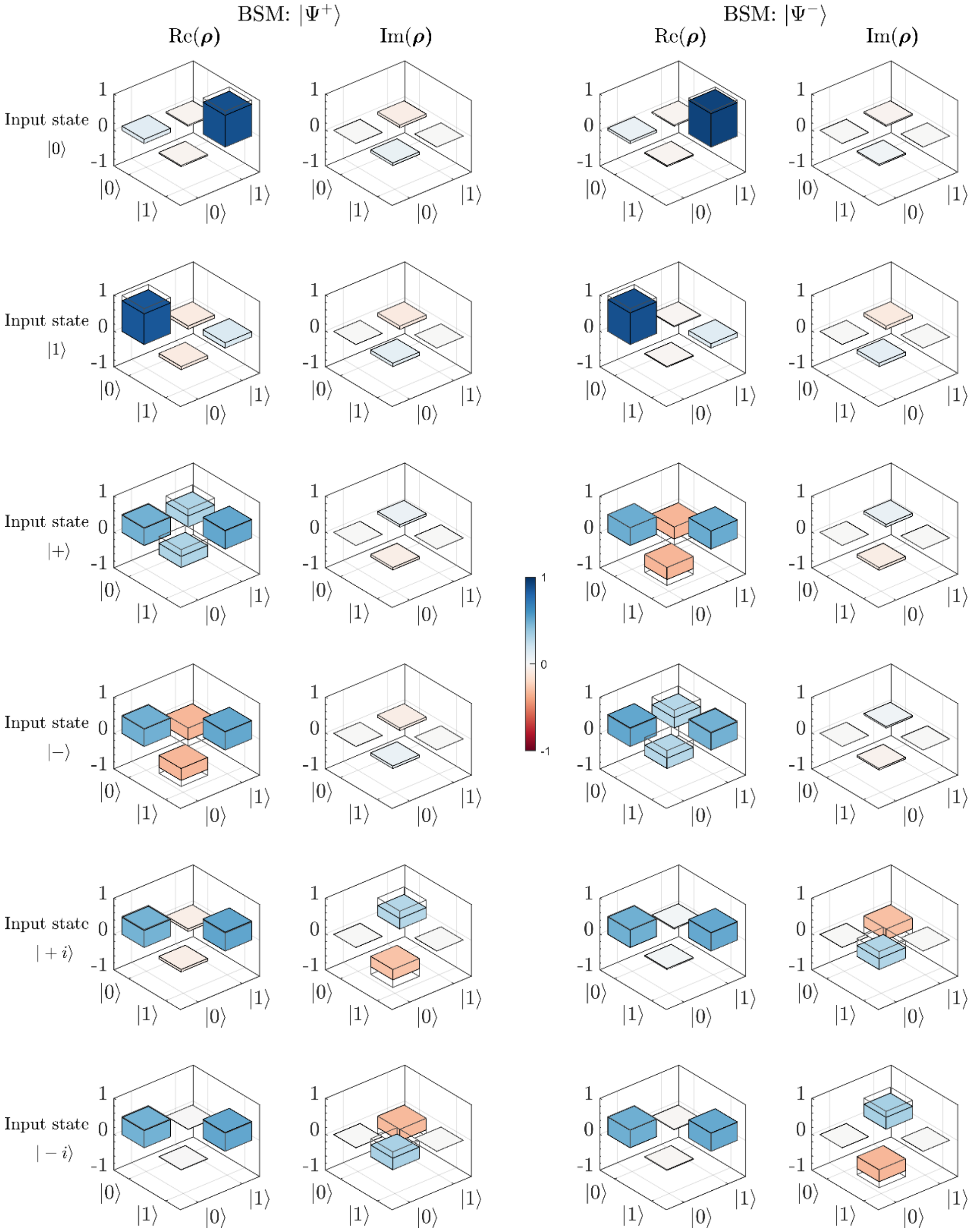

Fig. S11 Reconstructed density matrices of the teleported states in the experiment without optical fiber transmission.

Supplementary information accompanies the manuscript on the Light: Science & Applications website (http://www.nature.com/lsa)

Reference

1. Watanabe, H., Yamada, N. & Okaji, M. Linear Thermal Expansion Coefficient of Silicon from 293 to 1000 K. *Int. J. Thermophys.* **25**, 221–236 (2004).
2. Komma, J., Schwarz, C., Hofmann, G., Heinert, D. & Nawrodt, R. Thermo-optic coefficient of silicon at 1550 nm and cryogenic temperatures. *Appl. Phys. Lett.* **101**, 041905 (2012).
3. Wang, X., Shi, W., Vafaei, R., Jaeger, N. A. F. & Chrostowski, L. Uniform and sampled bragg gratings in SOI strip waveguides with sidewall corrugations. *IEEE Photonics Technol. Lett.* **23**, 290–292 (2011).
4. Wang, X., Grist, S., Flueckiger, J., Jaeger, N. A. F. & Chrostowski, L. Silicon photonic slot waveguide Bragg gratings and resonators. *Opt. Express* **21**, 19029–19039 (2013).
5. Oser, D. *et al.* Coherency-Broken Bragg Filters: Overcoming On-Chip Rejection Limitations. *Laser Photonics Rev.* **13**, 1800226 (2019).
6. Wang, J. *et al.* On-chip ultra-high rejection and narrow bandwidth filter based on coherency-broken cascaded cladding-modulated gratings. *Photonics Res.* **12**, 979 (2024).
7. Harris, N. C. *et al.* Integrated source of spectrally filtered correlated photons for large-scale quantum photonic systems. *Phys. Rev. X* **4**, 041047 (2014).
8. Oser, D. *et al.* High-quality photonic entanglement out of a stand-alone silicon chip. *Npj Quantum Inf.* **6**, 31 (2020).
9. Shu, Z. *et al.* On-chip superconducting nanowire single-photon detectors integrated with pump rejection for entanglement characterization. *Photonics Res.* **13**, 1067–1073 (2025).
10. Kaltenbaek, R., Blauensteiner, B., Żukowski, M., Aspelmeyer, M. & Zeilinger, A. Experimental Interference of Independent Photons. *Phys. Rev. Lett.* **96**, 240502 (2006).
11. Dou, T. *et al.* Coexistence of 1 Tbps classical optical communication and quantum key

distribution over a 100.96 km few-mode fiber. *Opt. Lett.* **48**, 4905–4908 (2023).

12. Burenkov, I. A. *et al.* Synchronization and coexistence in quantum networks. *Opt. Express* **31**, 11431–11446 (2023).

13. Rahmouni, A. *et al.* 100-km entanglement distribution with coexisting quantum and classical signals in a single fiber. *J. Opt. Commun. Netw.* **16**, 781–787 (2024).

14. Liu, J. *et al.* High-dimensional quantum key distribution using energy-time entanglement over 242 km partially deployed fiber. *Quantum Sci. Technol.* **9**, 015003 (2024).

15. Zukowski, M., Zeilinger, A. & Weinfurter, H. Entangling Photons Radiated by Independent Pulsed Sources. *Ann. N. Y. Acad. Sci.* **755**, 91–102 (1995).

16. Rarity, J. G. Interference of Single Photons from Separate Sources. *Ann. N. Y. Acad. Sci.* **755**, 624–631 (1995).

17. Marcikic, I., de Riedmatten, H., Tittel, W., Zbinden, H. & Gisin, N. Long-distance teleportation of qubits at telecommunication wavelengths. *Nature* **421**, 509–513 (2003).

18. De Riedmatten, H. *et al.* Long Distance Quantum Teleportation in a Quantum Relay Configuration. *Phys. Rev. Lett.* **92**, 047904 (2004).

19. Franson, J. D. Bell inequality for position and time. *Phys. Rev. Lett.* **62**, 2205–2208 (1989).

20. Marcikic, I. *et al.* Time-bin entangled qubits for quantum communication created by femtosecond pulses. *Phys. Rev. A* **66**, 062308 (2002).

21. Zhang, Z. *et al.* High-performance quantum entanglement generation via cascaded second-order nonlinear processes. *Npj Quantum Inf.* **7**, 123 (2021).
